# Cross-sectional Survey of Medical student perceptions of And desires for Research and Training pathways (SMART): an analysis of prospective cohort study of UK medical students

**DOI:** 10.1186/s12909-023-04881-2

**Published:** 2023-12-15

**Authors:** Gokul Parameswaran, Amelia Bowman, Catherine Swales, Setthasorn Zhi Yang Ooi, Shie Wei Chan, Priya Rose Babu, Daniele Ramsay, Sofia Kostoudi, Soham Bandyopadhyay, SMART-NANSIG-InCiSion Collaborative, Adele Mazzoleni, Artemis Mantzavinou, Rahma Hegy, Joseph Nicholson, Abigail Hainsworth, Natalie Wheelhouse, Emily Boyd, Mohammad Mofatteh, Namrata Juneja, Rahul Ganguly, Jashan Selvakumar, Prethy Kannadasan, Roshni Patel, Alexandra Aspinall, Ffinian Jones, Craig Liddell, Prerna Khanna, Lucas Ho, Tanzil Rujeedawa, Adil Iqbal, Bilal Amin, Jacob Tan, Tasnima Zaman Khan, Maddie Cobbin, Shekinah Osuchukwu, Shivani Pedda Venkatagari, Megan Richardson, Dorota Duklas, Owain Ellis, Vaibhavee Patel, Adithi Randeni, Tina Limbu, Priya Patel, Annabelle Lim

**Affiliations:** 1 Medical Sciences Division, University of Oxford, Oxford, UK; 2 Cardiff University School of Medicine, Cardiff, UK; 3 Imperial University School of Medicine, London, UK; 4 Imperial Brain and Spine Initiative, Imperial College London, London, UK; 5 Department of Brain Sciences, Imperial College London, London, UK; 6 School of Medicine, University of St Andrews, North Haugh, St Andrews, UK; 7 Clinical Neurosciences, Clinical & Experimental Sciences, Faculty of Medicine, University of Southampton, Southampton, Hampshire UK; 8 Wessex Neurological Centre, University Hospital Southampton NHS Foundation Trust, Southampton, UK; 9 Department of Surgical Sciences, Oxford University Global Surgery Group, University of Oxford, St. Hilda’s College, NuffieldOxford, OX4 1DY UK

**Keywords:** Clinical academia, Medical students, Career progression, Collaborative study

## Abstract

**Objective:**

Clinician-scientists are critical to medical innovation and research. However, the number of clinician scientists in the UK has been declining steadily over the last decade. One of the cited reasons is poor student recruitment to academic training pathways. The SMART study aims to assess current student perceptions on research and identify key factors influencing whether a student is interested in research.

**Design:**

We conducted a cross-sectional survey study between January and May 2022.

**Setting:**

This was a multi-centre national study with data collected across 40 universities offering medical courses in the UK.

**Participants:**

Participants were UK medical students enrolled in medicine for 21/22 academic year.

**Main outcome and measure:**

The main outcomes were related to participant perceptions on research and whether they were interested in engaging with research in their future career. These measures were correlated with demographic and non-demographic details using regression analyses.

**Results:**

One thousand seven hundred seventy-four individuals participated in the SMART survey from 40 medical schools. Nearly half the participants felt there were barriers preventing them from doing research (46.67%) and almost three-quarters felt it was at least somewhat difficult to combine research with medical school (73.49%). Of the options available, most commonly students did not want to pursue an academic career (43.11%) or training pathway (42.49%). However, most participants felt it was useful to do research at medical school (59.54%) and were also interested in doing more research in the future (69.16%). Regression analysis identified many factors influencing student’s perceptions of research including year of study, gender, socioeconomic status, family background, research exposure at medical school, ethnicity, and country of pre-university education.

**Conclusions:**

The SMART study is the first of its kind in the UK, shedding light on medical student perceptions. While some express strong interest in academic careers, a larger proportion show a broader interest in research. Demographic factors like gender, parental occupation, and socioeconomic status play a role. Further exploration is needed for specific groups to address barriers, promote research, and boost academic pathway recruitment.

**Supplementary Information:**

The online version contains supplementary material available at 10.1186/s12909-023-04881-2.

## Introduction

Clinician-scientists combine discovery science with clinical practice [1], and have an integrative outlook on medical science to address pressing healthcare issues, allowing them to make critical advancements in medicine and incorporate them into clinical practice as effectively as possible [2, 3]. Indeed, some of the most notable advancements in medicine have come from clinician-scientists with 37% of Nobel Prize Winners in Physiology and Medicine holding MD or equivalent qualifications [4]. However, recent studies suggest a decline in the number of clinician-scientists in the UK [5]. One of the reasons cited for this is an insufficient number of individuals entering the ‘clinician-scientist pipeline’ to replace an ageing workforce [5]. This is despite the UK's long history of enabling clinical-research training programs as well as it being a General Medical Council (GMC) requirement for medical students to be exposed to research. Given the need to maintain and ideally increase the number of clinician-scientists, it is critical to identify the key factors determining whether medical students choose to pursue research and academic pathways.

Studies conducted in other countries suggest medical students face several challenges in gaining research experience including lack of time, lack of funding and lack of formal teaching of research methodology [6–8]. These barriers seem to persist in even later training as American medical residents report insufficient time and inadequate research skills to conduct research despite feeling it should be a requirement [9]. Other studies also suggest intrinsic demographic factors such as ethnicity, gender, and economic background affect interest in academic training pathways [10, 11]. However, the relatively small sample size of these studies has prevented definitive associations from being drawn. Furthermore, no similar data about student attitudes towards research exists for the UK. Given the differences in medical school requirements for research as well as, arguably, a better-defined clinician-scientist training pathway, it is critical to understand different attitudes of UK medical students towards both—a career in clinical academia and research more broadly.

Therefore, a large-scale prospective survey of a convenience sample of medical students in the UK was conducted to investigate medical student perceptions and determine the interactions between demographic and non-demographic factors on attitudes towards research using an online national questionnaire called ‘Survey of Medical student Attitudes to Research and Training pathways (SMART)’. The SMART study aims to better understand the barriers deterring students from pursuing academic careers and encourage solutions to foster the next generation of clinician-scientists.

## Methods

The study protocol has been previously published [12], and all deviations from the protocol are noted below.

### Participants & recruitment

Participants were eligible for this study if they were medical students in the UK undertaking a medical course in one of 42 institutions recognised by the General Medical Council (GMC). Students from all years of both graduate-entry and undergraduate medical courses were eligible. Students were included from their first year, as some first-year medical students have completed prior academic degrees, which may have involved research experience. Furthermore, other first-year students may have taken a gap year, during which they could have had the opportunity to engage in research activities. Admission processes for medical schools often require students to discuss research, either referenced in their personal statements or during interviews. This further supports the argument that even first-year students will have some level of familiarity with research concepts. Complementing this, the GMC stipulates all UK medical students should have exposure to research annually. Our data collection began in January, therefore first-year students should have encountered research in their first term, as per GMC guidelines. In addition to these points, the aim of our study is to explore medical student perceptions and attitudes toward research across a wide educational spectrum and research exposure. Including only final-year students would have limited the scope and potentially skewed the results, as these students have likely solidified their career plans.

Recruitment was conducted through various routes including social media, medical school societies, academic medical organisations, conferences, the national network of INSPIRE leads [13] (a member of faculty nominated by the Head/Dean of UK Medical School who has overall responsibility for work funded by the Academy of Medical Sciences) and medical school mailing lists. Fresher’s fairs – an event introducing the universities’ societies to new students – as listed in the original protocol, were not utilised due to the time of year of data collection. To aid in disseminating the questionnaire amongst medical students, each university had at least one regional lead responsible for distributing the study amongst students at their institution. Regional leads were kept informed of updates and numbers by study leads.

### Questionnaire design

The questionnaire (Additional file 1: Appendix S1) followed the guidelines of the Association for Medical Education in Europe for questionnaire development [14]. The main study instrument was an online semi-qualitative survey comprising a total 24 questions, rather than 23 questions as originally described [12], utilising a combination of a 5-point Likert scale, multiple choice questions and free text. The survey aimed to uncover factors influencing medical school research participation, including demographic and non-demographic aspects. The study instrument underwent expert validation through the UK Medical Schools Council (MSC) Education Lead Advisory Group. Online questionnaires were completed using Qualtrics software (cloud-based platform capable of forming/modifying databases) compliant with GDPR and ICH E6 Good Medical Practice regulations. An internal pilot study was conducted, and the data from the internal pilot was carried forward towards the main national study. The original dates for data collection (November 2021 to January 2022) were delayed with study enrolment taking place from January to May 2022. Approval for direct dissemination to medical schools was obtained from the MSC. Additionally, we offered financial incentives in the form of Amazon vouchers to four randomly selected respondents.

### Primary outcome & secondary outcomes

The primary outcome was ‘to describe the extent of current medical students' involvement with research’. In this study, the term research is used to encapsulate various scholarly endeavours. These range from quality improvement projects and clinical audits, often aimed at improving healthcare practices, to basic science projects that are laboratory focused. We also consider contributions to peer-reviewed and non-peer-reviewed publications as a form of research involvement. Furthermore, presentations at academic forums are included as they represent a dissemination of research findings. The term aims to capture the diverse avenues through which medical students can be involved in academic research. This was measured using the following variables: amount of research undertaken (ordinal); reasons for doing research (nominal); whether research was a compulsory part of the degree (binary); types of research undertaken (nominal); and perception of how well their medical school has educated them about research (ordinal).

The secondary outcomes were ‘to identify and understand the reasons driving and excluding students from research’ and ‘to identify factors that might encourage current students to conduct research’. This was assessed by correlating demographic details with 6 outcome measures – student wanting to pursue an academic career (ordinal), student wanting to pursue an academic pathway (ordinal), student wanting to undertake more research in the future (ordinal), difficulty in combining research with medical studies (ordinal), usefulness of research in combination with medical studies (ordinal) and perception of barriers preventing research engagement (nominal). The independent variables influencing student perception of research considered in this study were year in medical school (ordinal), previous academic degrees (nominal), prior involvement with research (as part of primary outcome), ethnic background (nominal), gender identity (nominal), LGBTQ + status (binary), socioeconomic status (binary), family background (ordinal), and area of pre-university education (nominal).

### Sample size

In determining the prospective sample size, we conducted a sample size calculation based on survey methodology. Using a 95% confidence level and a margin of error of ± 5%, and assuming a prevalence rate of 50% for the variable of interest, it was calculated a minimum of 382 participants would be needed for statistically meaningful results. This calculation was adjusted for a finite population of 52,000 medical students. To account for dropouts or incomplete responses, we aimed to exceed this minimum number. Our recruitment strategy targeted students from all 42 accredited UK medical schools to reduce selection bias and enhance generalisability of findings. Given each additional participant beyond the minimum required would incrementally contribute to increased accuracy, we set a target of obtaining at least 1000 responses.

### Statistical analysis

Anonymised data was exported from Qualtrics. We used a 5% level of significance, and we provided 95% confidence intervals for our estimates. Firstly, data was pre-processed and cleaned by removing incomplete/duplicate entries. Data was deemed ‘incomplete’ if none of the outcome measures were answered. Some initial entries were duplicated as we tested the survey was collecting responses as expected. These were noted when responses were entered and were removed during the data pre-processing stage of our statistical analysis.

*Quantitative data:* comparison of proportions between groups was made with χ2. Univariate and ordinal logistic regression were used to examine the association between outcomes and participant characteristics. Multivariable logistic regression was used to explore effect of factors that had at least weak evidence for significance (p < 0·10) within the univariate analyses. Bonferroni correction was applied to variables that were significant on univariate regression in the multivariable regression to account for the multiple comparisons being made in this study. Covariates included year of medical school, previous academic degrees, and demographic information to control for potential confounding effects. All data analysis was done using STATA 16.1 and Python 3.9.1.

*Qualitative data:* Braun and Clarke’s reflexive thematic analysis was used to analyse qualitative responses [15]. All authors familiarised themselves with all data, creating initial inductive, descriptive codes. To identify themes, a semantic approach was used, analysing initial codes for patterns, then grouping, summarising, and interpreting themes. Author GP checked themes and subthemes against initial codes and data, presenting any interpretative discrepancies to the group. All authors then discussed and agreed themes and subthemes. To explore how the conceptual framework of self-regulated learning applied to involvement with research, we used a theory-informing inductive analytical approach [16]. To align with this approach, we first inductively analysed all data without imposing theoretical insights. Once quantitative data were analysed, and qualitative data had been grouped into preliminary categories, we then applied the conceptual framework of self-regulated learning as a 'sensitising concept' [17]. Authors reviewed all original data, codes, and themes alongside this framework, exploring and discussing areas of concordance and conflict. This contributed to the definition of our themes, and the discussion of this study.

### Ethics & funding

Ethics approval was obtained from the Medical Sciences Interdivisional Research Ethics Committee, Oxford, England (reference R73479/RE001). This work was supported by an INSPIRE grant from the Academy of Medical Sciences, with extension granted because of delays due to the COVID-19 pandemic.

## Results

### Demographics

 After cleaning the initial dataset of 2182 responses, we analysed 1772 responses (81.21%). This represented 95% (40/42) of UK medical schools listed by the MSC for 2021/22 and 4.2% of the total UK medical student population. Of the total eligible participants, 1566 were on an undergraduate (88.37%) and 206 were on a graduate-entry course (11.63%). Most participants had completed their pre-university education in the UK (80.70%) and did not have a prior academic degree (63.09%). They were also mostly female (65.52%), white (50.96%), did not identify themselves as members of the LBGTQ + community (83.18%), had not been on school meals (88.21%) and had no first-degree relatives in healthcare (54.76%), academia (54.18%) or academia AND healthcare (77.93%). This data is fully summarised in Table 1.

**Table 1:** Demographic details of SMART study participants

Characteristics		Number (%) of participants
*Type of medical course*	Undergraduate	1566 (88.37)
	Graduate Entry	206 (11.63)
Year of medical education (undergraduate entry*)	1^st^ Year	269 (17.18)
	2^nd^ Year	314 (20.05)
	3^rd^ Year	302 (19.41)
	4^th^ Year	289 (18.45)
	5^th^ Year	230 (14.69)
	Intercalations	162 (10.34)
Year of medical education (graduate entry*)	1^st^ Year	46 (21.30)
	2^nd^ Year	38 (17.59)
	3^rd^ Year	63 (29.17)
	4^th^ Year	63 (29.17)
	Intercalations	6 (2.78)
*Region of pre-university education*	UK	1430 (80.70)
	EU	85 (4.80)
	Others	255 (14.39)
	Missing	2 (0.11)
*Previous academic degrees*	Yes	649 (36.63)
	No	1118 (63.09)
	Missing	5 (0.28)
*Gender identity*	Male	575 (32.45)
	Female	1161 (65.52)
	Non-Binary	20 (1.13)
	Prefer Not to Say	9 (0.51)
	Others	3 (0.17)
	Missing	4 (0.23)
*LGBTQ* + *identity*	Yes	283 (15.97)
	No	1474 (83.18)
	Missing	15 (0.85)
*Ethnicity*	White	903 (50.96)
	British	720 (40.63)
	Irish	59 (3.33)
	Gypsy	1 (0.06)
	Others White	123 (6.94)
	Asian/Asian British	639 (36.06)
	Indian	221 (12.47)
	Pakistani	109 (6.15)
	Bangladeshi	41 (2.31)
	Chinese	113 (6.38)
	Arab	57 (3.22)
	Others	98 (5.53)
	Mixed/Multiple Ethnic groups	90 (5.08)
	White and Asian	45 (2.54)
	White and Black African	13 (0.73)
	White and Black Caribbean	6 (0.34)
	Others Mixed	26 (1.47)
	Black/African/ Caribbean/Black British	77 (4.35)
	African	62 (3.50)
	Caribbean	9 (0.51)
	Others	6 (0.34)
	Other Ethnic Groups	41 (2.31)
	Prefer Not to Say	22 (1.24)
*School meals eligibility*	Yes	209 (11.79)
	No	1563 (88.21)
*Number of first-degree relatives in healthcare*	None	1059 (54.76)
	One	360 (20.32)
	Two	221 (12.47)
	Three	46 (2.60)
	More than three	42 (2.37)
	Missing	44 (2.48)
*Number of first-degree relatives in academia*	None	960 (54.18)
	One	304 (17.16)
	Two	248 (14.00)
	Three	111 (6.26)
	More than three	99 (5.59)
	Missing	50 (2.82)
*Number of first-degree relatives in academia AND healthcare*	None	1,381 (77.93)
	One	203 (11.46)
	Two	81 (4.57)
	Three	21 (1.19)
	More than three	36 (2.03)
	Missing	50 (2.82)

### Medical student perceptions on research

Most commonly, study participants felt they had undertaken ‘a little’ research at medical school (47.80%) and medical school had educated them to a ‘somewhat adequate’ level (37.92%) about research. Most students also reported needing to conduct at least some research as a part of their degree (62.36%). Mandatory research was not associated with how much research a student felt they had done (χ^2^ = 3.71, *p* = 0.294), but there was an association between undertaking research as a compulsory part of a degree and how well they felt medical school educated them about research (χ^2^ = 40.14, *p* < 0.001). Students perceived they had been better educated about research if it was a compulsory component of their medical degree (OR: 2.09, CI: 1.65–2.65, *p* < 0.001, Bonferroni *p* < 0.01).

Students reported the most typically undertaken research activities were oral presentations (37.53%), poster presentations (36.57%) and basic science projects (35.33%). The most significant factors driving research participation were interest in the subject being researched (48.81%), career progression (40.69%) and personal development (40.69%). Of 144 responses citing other reasons, only 112 responded to the free text option. Of these, 85 responses (75.89%) reported they only undertook research as it was a compulsory part of their degree. No other common theme could be identified in the other free text responses. Appendices S2, S3, and S4 show the different research activities as well as a students’ motivation for engaging in research in the context of self-reported amounts of research participants feel they have done.

Almost half of the study participants (46.67%) felt there were barriers preventing them from getting involved with research. Of the 942 qualitative responses detailing these barriers, the most cited themes were inadequate signposting of opportunities (30.79%), time constraints (25.48%), a wider lack of research opportunities (15.07%) and insufficient opportunities to network with researchers (13.80%). Less commonly cited reasons were lack of funding (5.20%), limitations due to the pandemic (2.02%), and excess competition for research opportunities (1.70%) (Additional file 5: Appendix S5). Most participants felt it was at least ‘somewhat difficult’ to combine research with medical studies (73.49%). However, most respondents felt it was ‘moderately useful’ (32.28%) or ‘very useful’ (27.26%) to do research along with their studies. With regards to future career plans, most commonly, participants did not want to pursue an academic career (43.11%) or training pathway (42.49%). However, most participants were ‘somewhat’ or ‘strongly’ interested (69.16%) in pursuing more research in the future. This information on student perceptions has been summarised in Table 2.

**Table 2:** Student perceptions of research exposure and attitudes

Question	Response Options	Number (%) of participants
*Level of participants' exposure to research to date*	None at All	356 (20.09)
	A Little	847 (47.80)
	A Moderate Amount	434 (24.49)
	A Lot	97 (5.47)
	A Great Deal	36 (2.03)
	Missing	2 (0.11)
*Adequacy of medical school’s contribution to participants' knowledge of research*	Extremely inadequate	116 (6.55)
	Somewhat inadequate	449 (25.34)
	Neither adequate nor inadequate	353 (19.92)
	Somewhat adequate	672 (37.92)
	Extremely adequate	181 (10.21)
	Missing	1 (0.06)
*Research compulsory part of a degree*	Yes	1105 (62.36)
	No	283 (15.97)
	Missing	384 (21.67)
*Reasons for engaging with research*	Interest in the subject	865 (48.81)
	Career progression	721 (40.69)
	Personal development	717 (40.46)
	Interest in scientific problems	596 (33.63)
	Feel obliged to do it	520 (29.35)
	Contribution to better health care	465 (26.24)
	Intellectual stimulation	437 (24.60)
	Improving critical thinking	384 (21.67)
	Extra Income	89 (5.02)
	Other	144 (8.13)
	Missing	2 (0.11)
*Types of research done*	Quality Improvement Project	268 (15.12)
	Audit	425 (23.98)
	Basic Science Project	626 (35.32)
	Co-author on Original Paper in Peer Reviewed Journal	207 (11.68)
	Co-author on any Publications Related to Research	181 (10.21)
	Presented a Poster	648 (36.57)
	Given an Oral Presentation	665 (37.53)
	Named Collaborator on Original Paper in Peer Reviewed Journal	99 (5.59)
	Named Collaborator on any Publication Related to Research	112 (6.32)
	Clinical Project	459 (25.90)
	Other	137 (7.73)
	Missing	2 (0.11)
*Barriers preventing research*	Yes	827 (46.67)
	No	928 (52.37)
	Missing	17 (0.96)
*Usefulness of research with medical studies*	Not at all useful	70 (3.95)
	Slightly useful	422 (23.81)
	Moderately useful	572 (32.28)
	Very useful	483 (27.26)
	Extremely useful	216 (12.19)
	Missing	9 (0.54)
*Difficulty of combining research with medical studies*	Extremely difficult	286 (16.14)
	Somewhat difficult	963 (54.35)
	Neither easy nor difficult	348 (19.64)
	Somewhat easy	137 (7.73)
	Extremely easy	21 (1.19)
*‘I wish to pursue an academic career’*	Strongly disagree	310 (17.49)
	Somewhat disagree	454 (25.62)
	Neither agree nor disagree	407 (22.97)
	Somewhat agree	422 (23.81)
	Strongly agree	178 (10.05)
	Missing	1 (0.06)
*‘I wish to pursue an academic training pathway’*	Strongly disagree	334 (18.85)
	Somewhat disagree	443 (25.00)
	Neither agree nor disagree	427 (24.10)
	Somewhat agree	388 (21.90)
	Strongly agree	177 (9.99)
	Missing	3 (0.17)
*‘I would be interested in undertaking more research in the future’*	Strongly disagree	119 (6.72)
	Somewhat disagree	208 (11.74)
	Neither agree nor disagree	248 (14.00)
	Somewhat agree	660 (37.25)
	Strongly agree	530 (29.91)
	Missing	7 (0.40)

### Participant’s perceived barriers to research

Several factors influenced students' perceptions of research and their likelihood of pursuing an academic career. Table 3 presents the significant results from regression analyses (refer to Additional file 6: Appendix S6 for all analyses performed). Gender was significant, with women having higher odds of perceiving greater barriers to combining research with medical studies compared to men (OR: 1.968; *p* < 0.001; Bonferroni *p* < 0.01), despite their higher odds of interest in future research engagement (OR: 1.345; *p* = 0.012; Bonferroni *p* = 0.10). Students' perceptions were also influenced by the career paths of their immediate family members. Participants with first-degree relatives in academia had lower odds of wanting to pursue research in the future (OR 0.912; *p* = 0.011; Bonferroni *p* = 0.09). Asian ethnicity was also associated with a higher chance of desiring an academic career (OR 1.308; *p* = 0.033; Bonferroni *p* = 0.26).

**Table 3:** Factors influencing students’ perceptions of research and their likelihood of pursuing an academic career

		Univariate ordinal logistic regressiona			Multivariable ordinal logistic regression		
		OR (95% CI)	*P*-value	Bonferroni *P*-value	OR (95% CI)	*P*-value	Bonferroni *P*-value
Medical School has well educated the individual about research							
Completed an academic degree	Yes	1.274 [1.068 – 1.520]	0.007	0.06	1.040 [0.850 – 1.273]	0.7	5.60
	No	Reference	-	-	-	-	-
Ethnicity	Other ethnic group	0.683 [0.469 – 0.996]	0.048	0.38	0.825 [0.536 – 1.270]	0.382	3.06
	White	Reference	-	-	-	-	-
Per first degree relative who has been a healthcare professional		1.088 [1.012 – 1.169]	0.022	0.18	1.100 [1.014 – 1.193]	0.021	0.17
Area where they undertook most of their pre-university education	Outside the EU	0.588 [0.463 – 0.748]	< 0.001	< 0.01	0.566 [0.423 – 0.757]	< 0.001	< 0.01
	UK	Reference	-	-	-	-	-
Research compulsory part of a degree	Yes	2.092 [1.649 – 2.654]	< 0.001	< 0.01	2.014 [1.572 -2.581]	< 0.001	< 0.01
	No	Reference	-	-	-	-	-
Has undertaken research to date							
Year of medical school	3	2.001 (1.493 – 2.681)	< 0.001	< 0.01	2.001 [1.476 – 2.713]	< 0.001	< 0.01
	4	2.421 (1.794 – 3.269)	< 0.001	< 0.01	1.887 [1.377 – 2.585]	< 0.001	< 0.01
	5	3.153 (2.288 – 4.343)	< 0.001	< 0.01	2.284 [1.609 – 3.242]	< 0.001	< 0.01
	1	Reference	-	-	-	-	-
Type of degree	Post-graduate	1.689 [1.289 – 2.213]	< 0.001	< 0.01	1.060 [0.769 – 1.462]	0.721	5.77
	Undergraduate	Reference	-	-	-	-	-
Completed an academic degree	Yes	2.948 [2.447 – 3.551]	< 0.001	< 0.01	3.161 [2.500 – 3.997]	< 0.001	< 0.01
	No	Reference	-	-	-	-	-
Ethnicity	Other ethnic group	1.648 [1.114 – 2.437]	0.012	0.10	1.822 [1.190 – 2.790]	0.006	0.05
	White	Reference	-	-	-	-	-
Per first degree relative who has been a healthcare professional		1.088 [1.011 – 1.172]	0.025	0.20	1.044 [0.963 – 1.131]	0.296	2.37
Area where they undertook most of their pre-university education	EU	1.695 [1.128 – 2.549]	0.011	0.09	2.334 [1.484 – 3.669]	< 0.001	< 0.01
	Outside the EU	1.512 [1.182 – 1.934]	0.001	0.01	1.813 [1.354 – 2.428]	< 0.001	< 0.01
	UK	Reference	-	-	-	-	-
Adequacy of medical school’s contribution to participants' knowledge of research	Neither adequate nor inadequate	1.676 [1.124 – 2.500]	0.011	0.09	1.940 [1.258 – 2.993]	0.003	0.02
	Somewhat adequate	2.449 [1.678 – 3.574]	< 0.001	< 0.01	2.787 [1.853 – 4.191]	< 0.001	< 0.01
	Extremely adequate	5.059 [3.234 – 7.914]	< 0.001	< 0.01	5.822 [3.576 – 9.479]	< 0.001	< 0.01
	Extremely inadequate	Reference	-	-	-	-	-
Finds research useful in combination with medical studies							
Year of medical school	2	0.682 [0.517 – 0.899]	0.007	0.06	0.708 [0.535 – 0.936]	0.015	0.12
	3	0.545 [0.414 – 0.718]	< 0.001	< 0.01	0.530 [0.399 – 0.702]	< 0.001	< 0.01
	4	0.563 [0.426 – 0.744]	< 0.001	< 0.01	0.529 [0.397 – 0.703]	< 0.001	< 0.01
	5	0.610 [0.451 – 0.825]	0.001	0.01	0.561 [0.410 – 0.768]	< 0.001	< 0.01
	1	Reference	-	-	-	-	-
Area where they undertook most of their pre-university education	EU	1.978 [1.326 – 2.951]	0.001	0.01	1.760 [1.143 – 2.710]	0.01	0.08
	Outside the EU	1.320 [1.034 – 1.684]	0.026	0.21	1.268 [0.981 – 1.637]	0.069	0.55
	UK	Reference	-	-	-	-	-
Adequacy of medical school’s contribution to participants' knowledge of research	Extremely adequate	1.718 [1.106 – 2.670]	0.016	0.13	1.659 [1.032 – 2.667]	0.037	0.30
	Extremely inadequate	Reference	-	-	-	-	-
Research undertaken to date	A moderate amount	1.593 [1.235 – 2.053]	< 0.001	< 0.01	1.564 [1.180 – 2.073]	0.002	0.02
	A lot	2.356 [1.570 – 3.537]	< 0.001	< 0.01	1.911 [1.229 – 2.973]	0.004	0.03
	A great deal	5.116 [2.708 – 9.664]	< 0.001	< 0.01	3.768 [1.837 – 7.730]	< 0.001	< 0.01
	None at all	Reference	-	-	-	-	-
Finds combining research with medical studies difficult							
Year of medical school	2	0.754 [0.564 – 1.007]	0.056	0.45	0.836 [0.620 – 1.127]	0.239	1.91
	3	0.675 [0.505 – 0.903]	0.008	0.06	0.797 [0.589 – 1.079]	0.142	1.14
	4	0.674 [0.502 – 0.906]	0.009	0.07	0.759 [0.554 – 1.040]	0.087	0.70
	5	0.730 [0.529 – 1.006]	0.054	0.43	0.878 [0.621 – 1.242]	0.462	3.70
	1	Reference	-	-	-	-	-
Type of degree	Post-graduate	0.654 [0.495 – 0.862]	0.003	0.02	0.713 [0.517 – 0.984]	0.04	0.32
	Undergraduate	Reference	-	-	-	-	-
Completed an academic degree	Yes	0.808 [0.671 – 0.973]	0.024	0.19	0.767 [0.607 – 0.969]	0.026	0.21
	No	Reference	-	-	-	-	-
Gender	Female	0.718 [0.592 – 0.870]	0.001	0.01	0.700 [0.570 – 0.859]	0.001	0.01
	Non-binary/ third gender	0.278 [0.115 – 0.675]	0.005	0.04	0.250 [0.096 – 0.651]	0.005	0.04
	Male	Reference	-	-	-	-	-
Adequacy of medical school’s contribution to participants' knowledge of research	Neither adequate nor inadequate	2.159 [1.420 – 3.284]	< 0.001	< 0.01	1.937 [1.235 – 3.036]	0.004	0.03
	Somewhat adequate	2.388 [1.606 – 3.550]	< 0.001	< 0.01	2.082 [1.362 – 3.184]	0.001	0.01
	Extremely adequate	6.050 [3.797 – 9.639]	< 0.001	< 0.01	4.767 [2.885 – 7.878]	< 0.001	< 0.01
	Extremely inadequate	Reference	-	-	-	-	-
Research undertaken to date	A moderate amount	1.695 [1.289 – 2.230]	< 0.001	< 0.01	1.442 [1.053 – 1.974]	0.022	0.18
	A lot	2.185 [1.410 – 3.385]	< 0.001	< 0.01	1.607 [0.987 – 2.615]	0.056	0.45
	None at all	Reference	-	-	-	-	-
Usefulness of research with medical studies	Moderately useful	2.476 [1.497 – 4.094]	< 0.001	< 0.01	2.308 [1.302 – 4.090]	0.004	0.03
	Very useful	2.696 [1.622 – 4.480]	< 0.001	< 0.01	2.552 [1.433 – 4.543]	0.001	0.01
	Extremely useful	3.435 [1.983 – 5.952]	< 0.001	< 0.01	2.873 [1.544 – 5.346]	0.001	0.01
	Not at all useful	Reference	-	-	-	-	-
Wants to pursue an academic career							
Year of medical school	2	0.811 [0.620 – 1.062]	0.128	1.02	1.096 [0.772 – 1.556]	0.609	4.87
	3	0.629 [0.481 – 0.822]	0.001	0.01	0.854 [0.612 – 1.193]	0.355	2.84
	4	0.770 [0.586 – 1.012]	0.061	0.49	1.045 [0.745 – 1.465]	0.8	6.40
	5	0.599 [0.444 – 0.808]	0.001	0.01	0.727 [0.508 – 1.041]	0.082	0.66
	1	Reference	-	-	-	-	-
Ethnicity	Asian/Asian British	1.349 [1.121 – 1.624]	0.002	0.02	1.308 [1.022 – 1.675]	0.033	0.26
	White	Reference	-	-	-	-	-
Eligible for free meals at school	Yes	1.306 [1.011 – 1.688]	0.041	0.33	1.353 [0.982 – 1.864]	0.065	0.52
	No	Reference	-	-	-	-	-
Area where they undertook most of their pre-university education	EU	1.860 [1.244 – 2.782]	0.003	0.02	1.609 [0.976 – 2.653]	0.062	0.50
	Outside the EU	1.335 [1.054 – 1.692]	0.017	0.14	1.097 [0.807 – 1.491]	0.554	4.43
	UK	Reference	-	-	-	-	-
Research compulsory part of a degree	Yes	0.758 [0.600 – 0.959]	0.021	0.17	0.744 [0.574 – 0.964]	0.025	0.20
	No	Reference	-	-	-	-	-
Research undertaken to date	A moderate amount	1.436 [1.119 – 1.844]	0.004	0.03	1.246 [0.988 – 1.570]	0.063	0.50
	A lot	2.722 [1.801 – 4.114]	< 0.001	< 0.01	2.091 [1.368 – 3.196]	0.001	0.01
	A great deal	5.287 [2.827 – 9.887]	< 0.001	< 0.01	2.596 [1.280 – 5.267]	0.008	0.06
	None at all	Reference	-	-	-	-	-
Usefulness of research with medical studies	Slightly useful	2.367 [1.438 – 3.896]	0.001	< 0.01	2.121 [1.142 – 3.940]	0.017	0.14
	Moderately useful	9.927 [6.021 – 16.368]	< 0.001	< 0.01	3.934 [2.136 – 7.244]	< 0.001	< 0.01
	Very useful	9.927 [6.021 – 16.368]	< 0.001	< 0.01	9.266 [4.979 -17.244]	< 0.001	< 0.01
	Extremely useful	37.736 [21.853 – 65.164]	< 0.001	< 0.01	30.202 [15.3–5 – 59.600]	< 0.001	< 0.01
	Not at all useful	Reference	-	-	-	-	-
Difficulty of combining research with medical studies	Somewhat difficult	1.274 [1.005 – 1.615]	0.046	0.37	1.303 [0.961 – 1.766]	0.089	0.71
	Extremely easy	3.539 [1.485 – 8.435]	0.004	0.03	1.464 [0.558 – 3.840]	0.438	3.50
	Extremely difficult	Reference	-	-	-	-	-
Wants to pursue an academic training pathway							
Year of medical school	2	0.764 [0.585 – 0.998]	0.048	0.38	0.907 [0.688 – 1.198]	0.492	3.94
	3	0.573 [0.438 – 0.749]	< 0.001	< 0.01	0.687 [0.518 – 0.911]	0.009	0.07
	4	0.689 [0.523 – 0.907]	0.008	< 0.01	0.848 [0.635 – 1.134]	0.267	2.14
	5	0.393 [0.289 – 0.533]	< 0.001	< 0.01	0.454 [0.329 – 0.627]	< 0.001	< 0.01
	1	Reference	-	-	-	-	-
Type of degree	Post-graduate	1.370 [1.056 – 1.778]	0.018	0.14	1.384 [1.045 – 1.833]	0.023	0.18
	Undergraduate	Reference	-	-	-	-	-
Ethnicity	Asian/Asian British	1.312 [1.091 – 1.578]	0.004	0.03	1.296 [1.046 – 1.604]	0.018	0.14
	White	Reference	-	-	-	-	-
Area where they undertook most of their pre-university education	EU	2.167 [1.457 – 3.221]	< 0.001	< 0.01	1.608 [1.031 – 2.508]	0.036	0.29
	Outside the EU	1.289 [1.015 – 1.635]	0.037	0.30	1.114 [0.848 – 1.464]	0.437	3.50
	UK	Reference	-	-	-	-	-
Research undertaken to date	A moderate amount	1.378 [1.075 – 1.767]	0.012	0.10	1.339 [1.008 – 1.778]	0.044	0.35
	A lot	2.636 [1.732 – 4.012]	< 0.001	< 0.01	2.365 [1.485 – 3.766]	< 0.001	< 0.01
	A great deal	5.648 [2.925 -10.903]	< 0.001	< 0.01	4.185 [1.899 – 9.223]	< 0.001	< 0.01
	None at all	Reference	-	-	-	-	-
Usefulness of research with medical studies	Slightly useful	2.012 [1.239 – 3.269]	0.005	0.04	1.957 [1.145 – 3.347]	0.014	0.11
	Moderately useful	3.408 [2.113 – 5.498]	< 0.001	< 0.01	2.984 [1.755 – 5.074]	< 0.001	< 0.01
	Very useful	6.493 [3.996 – 10.550]	< 0.001	< 0.01	5.463 [3.186 – 9.367]	< 0.001	< 0.01
	Extremely useful	20.916 [12.328 – 35.486]	< 0.001	< 0.01	14.624 [8.158 – 26.214]	< 0.001	< 0.01
	Not at all useful	Reference	-	-	-	-	-
Difficulty of combining research with medical studies	Somewhat difficult	1.464 [1.153 – 1.860]	0.002	0.02	1.372 [1.058 – 1.779]	0.017	0.14
	Neither easy nor difficult	1.470 [1.113 – 1.941]	0.007	0.06	1.287 [0.950 – 1.744]	0.103	0.82
	Somewhat easy	1.941 [1.339 – 2.813]	< 0.001	< 0.01	1.099 [0.734 – 1.646]	0.646	5.17
	Extremely easy	4.121 [1.678 – 10.119]	0.002	0.02	2.290 [0.886 – 5.918]	0.087	0.70
	Extremely difficult	Reference	-	-	-	-	-
Interested in undertaking more research in the future							
Year of medical school	2	0.851 [0.645 – 1.123]	0.255	2.04	1.188 [0.821 – 1.720]	0.361	2.89
	3	0.703 [0.534 – 0.927]	0.012	0.10	0.998 [0.699 – 1.426]	0.993	7.94
	4	1.084 [0.821 – 1.433]	0.569	4.55	1.776 [1.229 – 2.566]	0.002	0.02
	5	0.948 [0.699 – 1.284]	0.728	5.82	1.236 [0.835 – 1.828]	0.29	2.32
	1	Reference	-	-	-	-	-
Completed an academic degree	Yes	1.317 [1.105 – 1.570]	0.002	0.02	1.194 [0.945 – 1.508]	0.138	1.10
	No	Reference	-	-	-	-	-
Gender	Female	1.215 [1.014 – 1.457]	0.035	0.28	1.345 [1.068 – 1.694]	0.012	0.10
	No	Reference	-	-	-	-	-
Per first degree relative who has been in academia		0.939 [0.889 – 0.992]	0.025	0.20	0.912 [0.850 – 0.979]	0.011	0.09
Area where they undertook most of their pre-university education	EU	2.562 [1.687 – 3.892]	< 0.001	< 0.01	2.132 [1.258 – 3.614]	0.005	0.04
	Outside the EU	1.499 [1.174 – 1.913]	0.001	0.01	1.276 [0.943 – 1.727]	0.114	0.91
	UK	Reference	-	-	-	-	-
Research compulsory part of a degree	Yes	0.687 [0.538 – 0.876]	0.002	0.02	0.638 [0.480 – 0.848]	0.002	0.02
	No	Reference	-	-	-	-	-
Adequacy of medical school’s contribution to participants' knowledge of research	Somewhat inadequate	0.672 [0.455 – 0.992]	0.046	0.37	0.780 [0.462 – 1.315]	0.35	2.80
	Neither adequate nor inadequate	0.516 [0.346 – 0.771]	0.001	0.01	0.660 [0.386 – 1.128]	0.129	1.03
	Somewhat adequate	0.645 [0.441 – 0.943]	0.024	0.19	0.677 [0.409 -1.120]	0.129	1.03
	Extremely inadequate	Reference	-	-	-	-	-
Research undertaken to date	A moderate amount	1.337 [1.037 – 1.723]	0.025	0.20	1.168 [0.912 – 1.497]	0.219	1.75
	A lot	1.521 [1.002 – 2.309]	0.049	0.39	0.994 [0.636 – 1.555]	0.98	7.84
	A great deal	2.904 [1.511 – 5.583]	0.001	0.01	1.292 [0.580 – 2.877]	0.531	4.25
	None at all	Reference	-	-	-	-	-
Usefulness of research with medical studies	Slightly useful	4.408 [2.644 – 7.349]	< 0.001	< 0.01	3.805 [1.995 – 7.258]	< 0.001	< 0.01
	Moderately useful	10.817 [6.497 – 18.011]	< 0.001	< 0.01	8.600 [4.532 – 16.318]	< 0.001	< 0.01
	Very useful	30.852 [18.266 – 52.108]	< 0.001	< 0.01	23.871 [12.377 – 46.039]	< 0.001	< 0.01
	Extremely useful	127.233 [70.382 – 230.005]	< 0.001	< 0.01	88.214 [41.962 – 185.448]	< 0.001	< 0.01
	Not at all useful	Reference	-	-	-	-	-
Feels there are barriers preventing them from getting involved in research							
Year of medical school	2	1.292 [0.946 – 1.764]	0.107	0.86	1.697 [1.093 – 2.636]	0.018	0.14
	3	1.600 [1.176 – 2.177]	0.003	0.02	1.984 [1.299 – 3.028]	0.002	0.02
	4	1.795 [1.308 – 2.463]	< 0.001	< 0.01	2.432 [1.580 – 3.743]	< 0.001	< 0.01
	5	1.978 [1.406 – 2.781]	< 0.001	< 0.01	2.776 [1.764 – 4.369]	< 0.001	< 0.01
	1	Reference	-	-	-	-	-
Gender	Female	1.926 [1.567 – 2.366]	< 0.001	< 0.01	1.968 [1.496 – 2.590]	< 0.001	< 0.01
	Male	Reference	-	-	-	-	-
Per first degree relative who has been a healthcare professional		0.845 [0.773 – 0.923]	< 0.001	< 0.001	0.902 [0.788 – 1.031]	0.13	1.04
Per first degree relative who has been in academia		0.890 [0.832 – 0.951]	0.001	0.01	0.928 [0.839 – 1.026]	0.145	1.16
Per first degree relative who has held an academic position in the healthcare environment		0.851 [0.768 – 0.944]	0.002	0.02	0.896 [0.767 – 1.048]	0.17	1.36
Research compulsory part of a degree	Yes	0.716 [0.550 – 0.931]	0.013	0.10	0.723 [0.526 – 0.993]	0.045	0.36
	No	Reference	-	-	-	-	-
Adequacy of medical school’s contribution to participants' knowledge of research	Neither adequate nor inadequate	0.344 [0.219 – 0.539]	< 0.001	< 0.01	0.567 [0.301 – 1.068]	0.079	0.63
	Somewhat adequate	0.304 [0.198 – 0.465]	< 0.001	< 0.01	0.394 [0.217 – 0.718]	0.002	0.02
	Extremely adequate	0.164 [0.098 – 0.274]	< 0.001	< 0.01	0.314 [0.156 – 0.630]	0.001	0.01
Extremely inadequate	Reference	-	-	-	-	-	
Research undertaken to date	A moderate amount	0.427	< 0.001	< 0.01	0.674 [0.507 – 0.898]	0.007	0.06
	A lot	0.436	< 0.001	< 0.01	0.904 [0.545 – 1.499]	0.695	5.56
None at all	Reference	-	-	-	-	-	
Difficulty of combining research with medical studies	Somewhat difficult	0.356 [0.266 – 0.476]	< 0.001	< 0.01	0.389 [0.268 – 0.565]	< 0.001	< 0.01
	Neither easy nor difficult	0.190 [0.135 – 0.268]	< 0.001	< 0.01	0.239 [0.151 – 0.377]	< 0.001	< 0.01
	Somewhat easy	0.093 [0.057 – 0.153]	< 0.001	< 0.01	0.134 [0.072 – 0.248]	< 0.001	< 0.01
	Extremely easy	0.118 [0.042 – 0.332]	< 0.001	< 0.01	0.118 [0.031 – 0.450]	0.002	0.02
Extremely difficult	Reference	-	-	-	-	-	

^a^Only variables that were significant on univariate regression are presented

Students who had undertaken more research had a higher likelihood of expressing a greater interest in doing future research and were more likely to consider an academic career pathway. Similarly, those who believed their medical school had better educated them about research were more likely to find research useful with their medical studies and find it less difficult to combine with their medical studies. Respondents who had previously completed an academic degree had lower odds of finding it difficult to combine with their medical studies (OR 0.767; *p* = 0.026; Bonferroni *p* = 0.21). Graduate-entry medical students independently were less likely to find it difficult to combine research with their studies (OR 0.713; *p* = 0.040; Bonferroni *p* = 0.32) and had higher odds of wanting an academic training pathway (OR 1.384; *p* = 0.023; Bonferroni *p* = 0.18). However, students who engaged in research as a compulsory part of their degree were less likely to want an academic career (OR 0.744; *p* = 0.025; Bonferroni *p* = 0.20) or future research opportunities (OR 0.638; *p* = 0.002; Bonferroni *p* = 0.02), despite being more likely to feel they had been better educated about research (OR 2.014; *p* < 0.001; Bonferroni *p* < 0.01) and less likely to have barriers to pursuing research (OR 0.723; *p* = 0.045; Bonferroni *p* = 0.36). The year of study in the undergraduate course also demonstrated some correlation with research perception, with students in their 2nd year or above having lower odds of finding research useful for their studies or wanting to pursue an academic career compared to first year students. Students in 3rd year and beyond were also more likely to perceive barriers to research.

## Discussion

This study serves as the first multi-centre national survey of UK medical student perceptions about research. Among students surveyed, approximately 10% of students were strongly interested in pursuing an academic career or training pathway. Almost half of the participants felt there were barriers preventing them from getting involved with research, with more than half finding it somewhat or very difficult to combine research with medical studies. Furthermore, as students progressed through medical school, they were less likely to find research useful for their studies or want to pursue an academic career. This suggests they may encounter discouraging aspects of a research-oriented profession as they gain more experience. To gain deeper insights into the specific factors influencing this transition, additional research may be warranted.

However, about 60% of participants found research useful along with their medical studies and close to 70% were interested in doing more research in the future. This suggests, despite only a relatively small number of students being firmly interested in academic careers, many more are interested in research more broadly. By better understanding and addressing perceived barriers stopping students from pursuing academic pathways, we may be able to increase recruitment to academic pathways as well as clinician research engagement more broadly.

### Demographic factors and research perceptions

Based on the regression analysis we identified several demographic factors that influence a student’s perception on research. One of the most significant was gender, with women perceiving there to be greater barriers to performing and combining research with their medical school studies than men, despite being more interested in undertaking research in the future. These findings likely represent wider inequality in the current research landscape with only 30% of authors on research papers being women, and significantly fewer female researchers being promoted to associate or full professors [18, 19]. These wider inequalities in the research landscape may limit the exposure of women medical students to role models and mentor figures at medical school. This suggests, especially as women were more interested in engaging with research in the future, if there was institutional change focusing on increasing women’s research participation, more women may choose academic career pathways. Another factor influencing student perceptions towards research was the career of immediate family members. Those with first-degree relatives in academia were less likely to pursue an academic career pathway. These results fit with other studies that show a student’s career aspirations are strongly influenced by parental occupation [20, 21]. It is possible that students are being deterred from academia by reports of toxicity in academia by their parents [22].

We also found weak evidence for students of lower socioeconomic status (SES), indicated by eligibility for school meals, being more likely to want to pursue an academic career. This is consistent with previous reports showing students of lower SES making more active choices to pursue research opportunities such as intercalated degrees or summer research projects despite financial burdens [23]. This may be due to increased accessibility of grants and bursaries, but other reports suggest lower SES students are attracted to academic careers due to potential monetary rewards, prestige or even competitiveness [24, 25]. Asian students were found to be more likely to want to pursue an academic career than other ethnicities. There have not been previously published reports of this association to our knowledge. While prior surveys have shown Black or Hispanic students perceive more barriers to research, these studies did not show Asian students as being more interested in academia [26]. We also found students who had completed their pre-university education outside the UK, especially in the EU, were more likely to find research useful and want to pursue research in the future. The factors driving these students are poorly characterised by the current literature. Overall, these findings relating to free school meals, Asian ethnicity, and students from outside the UK all need further probing with large-scale, prospective qualitative studies to better understand the exact motivations driving these groups towards academia.

### Non-demographic factors and research perceptions

The primary non-demographic factor identified as influencing student perceptions of research was the research exposure of students. Those who had conducted more research at medical school were more likely to value research, want to conduct research in the future, and pursue an academic career pathway. Similarly, those who felt their medical school had better educated them about research were more likely to value research and find it easier to combine it with their medical studies. Furthermore, those who had already completed a previous academic degree were more likely to want to do more research in the future.

These findings, in line with previous reports, suggest students with prior research experience are more likely to want to work in academia [27, 28]. This indicates that should students be exposed to, educated about, and allowed to actively participate in research, it may cultivate the next generation of clinician-scientists. In line with this notion, students who needed to do research as a compulsory part of their degree felt better educated about research than those who did not. However, it also seems to be important that students are not overwhelmed with research. Our findings demonstrate this by showing a parabolic effect regarding the amount of research conducted and student’s view on research. Those who felt they had done very little, or a lot of research were both more likely to find it difficult to combine research with their studies and more likely to feel there were barriers to doing research than those who had done a moderate amount of research. Comparatively, students who had to complete research as a compulsory part of their course felt it was more difficult to combine it with their other medical studies. Furthermore, despite students from 3^rd^ year and above reporting conducting more research, they were generally more likely to perceive barriers to research and less likely to want to pursue an academic training pathway. Taken together these findings suggest that it may be crucial to expose students to research, but it is likely equally important to support and not overwhelm them such that research distracts from their core medical curriculum or takes too much time from other, non-academic pursuits. This may be especially important in the later years of medical school as students experience burnout [29].

### Study limitations

The study had several limitations. First, there was likely participation bias as medical students who already participate in research were more likely to engage with our survey. To reduce the impact of this, we distributed the survey through diverse modalities including directly via the medical school, using regional leads promoting the study directly to their peers as well as on social media. This led to a reduction in certain sources of bias, such as an equitable distribution of participants from undergraduate and graduate-entry medical courses that were similar to national Figs. [30]. Second, given our study being cross-sectional in nature, it limits our ability to explain causal relationships between variables. We are unable to understand how perceptions of surveyed students change over time and whether those interested in research at medical school chose to pursue it later in their careers. A longitudinal design would have allowed for an exploration of changes in attitudes of the respondents over time. Third, it is worth noting that the questionnaire did not explicitly test the medical students’ knowledge of medical research in terms of facts, methodologies, or principles, and therefore some participants may not have known the bounds and limitations of research. Fourth, individual assessments were conducted for each outcome. The use of multiple comparisons likely increased the likelihood of chance findings, as evident from the Bonferroni-adjusted p-values, which revealed that some of our initial findings lost their significance. Finally, it is worth noting the self-reported nature of the questionnaire meant there was likely some heterogeneity and subjectivity in the perception of the questions and response options by the participants. However, despite the lack of objectivity, the results are still valuable as it reflects current student attitudes.

## Conclusion

The SMART study is the first large-scale, multi-centre national survey comprehensively assessing medical student perceptions about research in the UK. We showed while a small percentage of students express a firm interest in academic careers, a larger proportion is interested in research more broadly. Our study highlights the influence of demographic factors such as gender, parental occupation, and socioeconomic status on students' research perceptions. Further research is needed to explore the motivations driving specific groups, such as students from lower SES, Asian backgrounds, and those educated outside the UK, toward academic careers. By addressing the perceived barriers and promoting research engagement, we can increase recruitment to academic pathways and enhance clinician research involvement.

### Supplementary Information

**Additional file 1. **

**Additional file 2: Appendix S2.** Cluster bar graph of the number of students who have done various types of research studies by their motivation to do research.

**Additional file 3: Appendix S3. **Cluster bar graph of the number of students who have presented research by their motivation to do research.

**Additional file 4: Appendix S4. **Cluster bar graph of the number of students who have authored a publication by their motivation to do research.

**Additional file 5: Appendix S5. **Individual Responses to the Free Text Questions.

**Additional file 6: Appendix S6. **Logistic regression results.

## Data Availability

The datasets used and/or analysed during the current study available from the corresponding author on reasonable request. Guarantor: Bandyopadhyay.
