# Supplementary material for: Cross-sectional Survey of Medical student perceptions of And desires for Research and Training pathways (SMART): an analysis of prospective cohort study of UK medical students
Source: BMC Med Educ. 2023 Dec 15;23:964. doi: 10.1186/s12909-023-04881-2 (PMC10725016; doi:10.1186/s12909-023-04881-2)
Supplement: Supplementary file 1 — Additional file 1. [file 12909_2023_4881_MOESM1_ESM.pdf]

Default Question Block

Cross-sectional Survey of Medical student Attitudes to Research and Training pathways (SMART) in the UK
CUREC Approval Reference: R73479/RE001

PURPOSE OF STUDY
This study aims to ascertain current medical student involvement with research. We also hope to identify factors encouraging and discouraging students from partaking in research and to consider what may encourage more engagement with scientific research in the future

WHY HAVE I BEEN CHOSEN?
You are being invited to take part in the questionnaire as you are a medical student currently studying for a UK medical degree at a UK medical school recognised by the General Medical Council (GMC)

WHAT DO I HAVE TO DO?
If you choose to participate in this voluntary survey, you will be asked to complete a questionnaire about your background, your previous exposure to research and your feelings towards a research career. This study is voluntary. If you decide not to participate this will not impact your academic standing in any way. If you decide to take part, you will be asked to complete the survey by clicking on the link below. This survey is expected to take about 10 -15 minutes to complete, but there is no time limit and you can take as much time as you like. No background knowledge is

required. We will ask for your consent for the collection and storage of data in accordance with the UK General Data Protection Regulation (GDPR) within the survey. For more information on GDPR please click on the following link: https://gdprinfo.eu/

DO I HAVE TO TAKE PART?
Please note that your participation is voluntary. You may withdraw at any point during the questionnaire for any reason, before submitting your answers, by closing the browser. In cases of withdrawal from the study, no new data will be collected or linked to other data from that point on. If you do not want to answer some of the questions you do not have to, but you can still be in the study. All questions are optional. Your decision whether or not to be part of the study will not affect your academic standing or your access to university support services. If you have already submitted data and wish to withdraw from the study, please contact soham.bandyopadhyay@st-hildas.ox.ac.uk by 1st August 2022.

WHAT ARE THE POSSIBLE BENEFITS OF TAKING PART?
Despite not have any immediate individual benefits by participating in this survey, you are given the opportunity to contribute to valuable and innovative research which could be used in the future by medical universities and the world. You may find this survey an opportunity to self-reflect. There will be the option to submit email address in order to be entered into a prize draw. At the conclusion of data collection, two random participants will be awarded £50 in Amazon vouchers, two further random participants will be awarded £25 in Amazon vouchers. This will be optional, as it requires you to provide personally identifying data (i.e. contact details). These will not be linked to the questionnaire answers given, and will only be used for contact regarding relevant rewards as above.

WHAT ARE THE POSSIBLE RISKS OF TAKING PART?
Some of the questions that we ask may cause upset. If you experience any distress from participating in this study, you may stop the survey at any time or skip any upsetting questions. If your distress continues after leaving the survey, we have provided a list of supportive services nationwide that can be helpful and that you might consider contacting.

HOW WILL MY DATA BE USED?
Your answers will be completely anonymous, and we will take all reasonable measures to ensure that they remain confidential. Your data will be stored in a password-protected file and may be used in academic publications. Your IP address will not be stored. If you provide us with your email address, we will delete that information at the end of the study. No answers will be linked to your email address. Research data – your anonymised answers – will be stored for a minimum of ten years after publication or public release.

WHO WILL HAVE ACCESS TO MY DATA?
Qualtrics is the data controller with respect to the personal data they hold about you and, as such, will determine how your personal data is used. Please see their privacy notice here: https://www.qualtrics.com/privacy-statement. Qualtrics will share

any email address you provide and your anonymised answers with the University of Oxford, for the purposes of research. Researchers involved in the project will have access to this anonymised data. The University of Oxford is the data controller of university email addresses, please see their privacy notice here: https://compliance.admin.ox.ac.uk/student-privacy-policy. Responsible members of the University of Oxford and funders may be given access to data for monitoring and/or audit of the study to ensure we are complying with guidelines, or as otherwise required by law.

WHAT WILL HAPPEN TO THE RESULTS OF THE RESEARCH PROJECT?
The findings of the study may be published in peer reviewed journals, presented at relevant conferences and meetings and a summary of the findings will be made available on social media.

WHO IS ORGANISING AND FUNDING THE RESEARCH?
Researchers at the University of Oxford. This study is funded by an INSPIRE grant from The Academy of Medical Sciences

WHO HAS REVIEWED THIS STUDY?
This project has received ethics clearance through the University of Oxford's ethical approval process for research involving human participants, reference R73479/RE001.

WHO DO I CONTACT IF I HAVE A CONCERN OR I WISH TO COMPLAIN?
If you have a concern about any aspect of this project, please speak to the researcher soham.bandyopadhyay@st-hildas.ox.ac.uk who will do their best to answer your query. The researchers should acknowledge your concern within 10 working days and give you an indication of how they intend to deal with it. If you remain unhappy or wish to make a formal complaint, please contact the Chair of the Research Ethics Committee at the University of Oxford who will seek to resolve the matter as soon as possible: Medical Sciences Interdivisional Research Ethics Committee; Email: ethics@medsci.ox.ac.uk; Address: Research Services, University of Oxford, Wellington Square, Oxford OX1 2JD

HOW DO I FIND OUT WHAT WAS LEARNT IN THIS STUDY?
This study is expected to be completed by approximately March 2022. If you would like a brief summary of the results, please write to us by email to request information

CONTACT FOR FURTHER INFORMATION
For any further questions or more information on the study, please contact us on the following email address: soham.bandyopadhyay@st-hildas.ox.ac.uk. Alternatively, you could contact principal investigator Dr Catherine Swales at catherine.swales@ndorms.ox.ac.uk.

Please note that you may only participate in this survey if you are 18 years of age or over.

○ I certify that I am 18 years of age or over

If you have read the information above and agree to participate with the understanding that the data (including any personal data) you submit will be processed accordingly, please check the relevant box below to get started.

○ Yes, I agree to take part

Please provide your email address if you would like to be considered for the prize draw, and are happy to be contacted via your email address for such

Block 1

Demographics

Which medical school do you attend? (If currently intercalating at a separate university, please give the university from which you will receive your main degree).

Which year of medical school are you currently in?

Are you on a graduate entry medical course?

○ Yes

○ No

Have you already completed an academic degree (Bachelor's/Masters/Doctorate)?

○ Yes

○ No

Please select all those degrees you currently have

- ☐ Bachelor's – in a scientific degree
- ☐ Bachelor's – in an arts degree
- ☐ Master's – in a scientific degree
- ☐ Master's – in an arts degree
- ☐ Doctorate
- ☐ Other

Choose one option that best describes your ethnic group or background?

- ○ White - British
- ○ White - Irish
- ○ White - Gypsy or Irish Traveller
- ○ White - Any other white background
- ○ Mixed/Multiple ethnic groups - White and Black Caribbean
- ○ Mixed/Multiple ethnic groups - White and Black African
- ○ Mixed/Multiple ethnic groups - White and Asian
- ○ Mixed/Multiple ethnic groups - Any other mixed/multiple ethnic background
- ○ Asian/Asian British - Indian
- ○ Asian/Asian British - Pakistani
- ○ Asian/Asian British - Bangladeshi
- ○ Asian/Asian British - Chinese
- ○ Asian/Asian British - Any other Asian/Asian British Background
- ○ Black/African/Caribbean/Black British - African
- ○ Black/African/Caribbean/Black British - Carribean
- ○ Black/African/Caribbean/Black British - Any other Black/African/Caribbean/Black British
- ○ Other ethnic group - Arab
- ○ Other
- ○ Prefer not to say

What best describes your gender?

- ○ Male
- ○ Female
- ○ Non-binary / third gender
- ○ Prefer not to say
- ○ Other

Do you identify as LGBTQ+?

- ○ Yes
- ○ No

During school, at any point, were you eligible for free school meals?

- ○ Yes
- ○ No

How many of your first degree relatives are or have been a healthcare professional?

How many of your first degree relatives are or have been in academia?

How many of your relatives hold or have held an academic position in the healthcare environment?

Block 2

Education

In which area did you undertake the majority of your pre-university education?

- ○ UK
- ○ EU
- ○ Outside the EU

How well do you feel your medical school has educated you about research?

- ○ Extremely inadequate
- ○ Somewhat inadequate
- ○ Neither adequate nor inadequate
- ○ Somewhat adequate
- ○ Extremely adequate

How much research have you undertaken to date?

- ○ None at all
- ○ A little
- ○ A moderate amount
- ○ A lot
- ○ A great deal

Was any of your research a compulsory part of a degree?

- ○ Yes
- ○ No

Please tick any of the following that apply to your research. You may tick more than one box or none at all

- ☐ Quality Improvement Project
- ☐ Audit
- ☐ Basic science project
- ☐ Clinical project
- ☐ Co-author on original paper in peer reviewed journal

- ☐ Co-author on any publications related to research
- ☐ Named collaborator on original paper in peer reviewed journal
- ☐ Named collaborator on any publication related to research
- ☐ Presented a poster
- ☐ Given an oral presentation
- ☐ Other

Are there barriers preventing you from getting involved with research?

- ○ Yes (please describe)
- ○ No

Why did/do you do research?

- ☐ Interest in scientific problems
- ☐ Interest in the subject
- ☐ Personal development
- ☐ Extra income
- ☐ Contribution to better health care
- ☐ Improving critical thinking
- ☐ Career progression
- ☐ Intellectual stimulation
- ☐ Feel obliged to do it
- ☐ Other

How useful do you think research is in combination with your medical studies?

- ○ Not at all useful
- ○ Slightly useful
- ○ Moderately useful
- ○ Very useful
- ○ Extremely useful

How difficult is it to combine research with your medical studies?

- ○ Extremely difficult
- ○ Somewhat difficult
- ○ Neither easy nor difficult
- ○ Somewhat easy
- ○ Extremely easy

How much do you agree with the following statements:

	I wish to pursue an academic career	I wish to pursue an academic training pathway	I would be interested in undertaking (more) research in the future
Strongly disagree	○	○	○
Somewhat disagree	○	○	○
Neither agree nor disagree	○	○	○
Somewhat agree	○	○	○
Strongly agree	○	○	○

What would encourage your involvement in research in the future?

- ○ More time
- ○ More incentives
- ○ Easier access to research groups and projects
- ○ Clearer information about how to get involved
- ○ Clearer information about benefits of research
- ○ Guaranteed rewards e.g. publication or presentation
- ○ Other

Survey Powered By Qualtrics
