# Supplementary material for: Cross-sectional Survey of Medical student perceptions of And desires for Research and Training pathways (SMART): an analysis of prospective cohort study of UK medical students
Source: BMC Med Educ. 2023 Dec 15;23:964. doi: 10.1186/s12909-023-04881-2 (PMC10725016; doi:10.1186/s12909-023-04881-2)

***Appendix S2 - Cluster bar graph of the number of students who have done various types of research studies by their motivation to do research. The height of each coloured bar represents the number of students who have done an element of research within that category. The title of each cluster relates to the participants’ responses to the question: “Why did/do you do research?” The title of each bar represents the respondents’ answer to the question: “How much research have you undertaken to date?”***


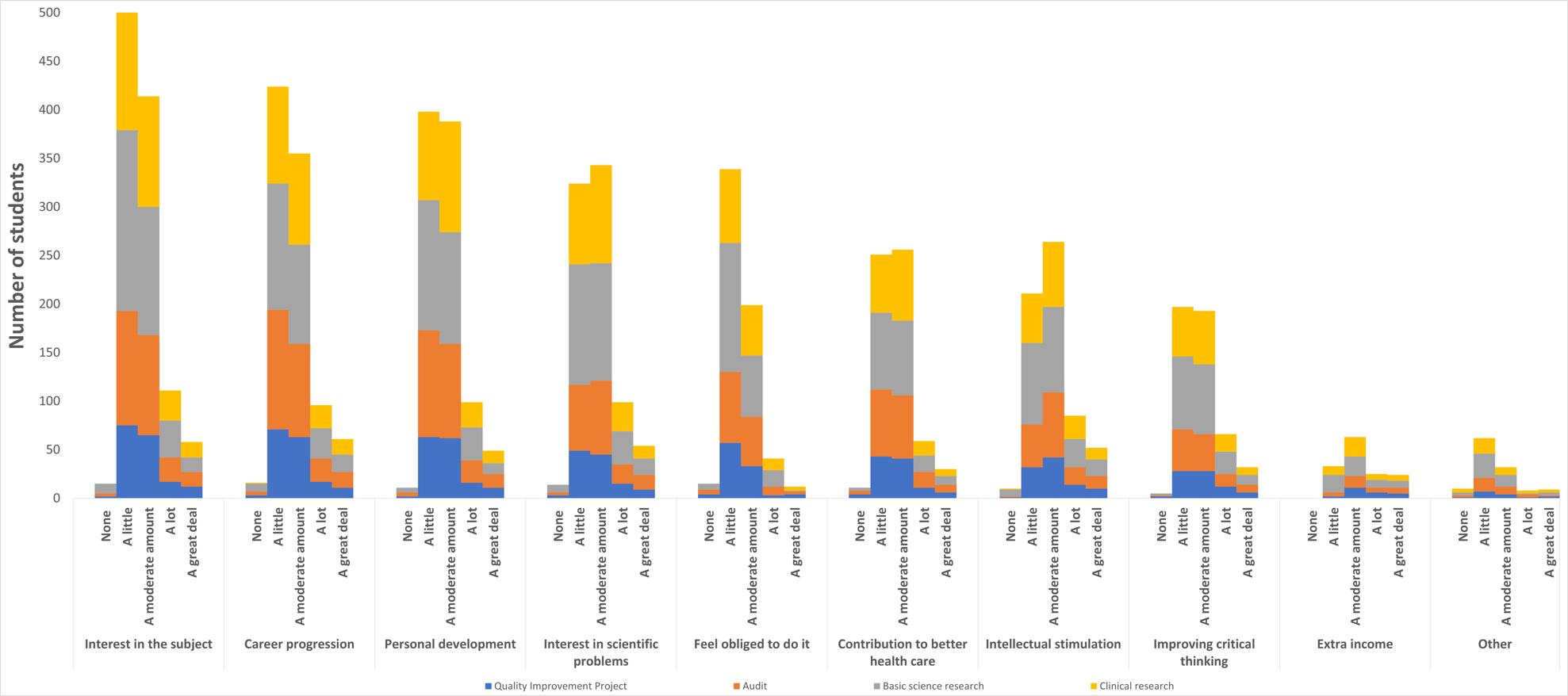

Supplement: Supplementary file 2 — Additional file 2: Appendix S2. Cluster bar graph of the number of students who have done various types of research studies by their motivation to do research. [file 12909_2023_4881_MOESM2_ESM.docx]
