# Supplementary material for: Cross-sectional Survey of Medical student perceptions of And desires for Research and Training pathways (SMART): an analysis of prospective cohort study of UK medical students
Source: BMC Med Educ. 2023 Dec 15;23:964. doi: 10.1186/s12909-023-04881-2 (PMC10725016; doi:10.1186/s12909-023-04881-2)

***Appendix S4 - Cluster bar graph of the number of students who have authored a publication by their motivation to do research. The height of each coloured bar represents the number of students who have either been co-author or collaborative author. The title of each cluster relates to the participants’ responses to the question: “Why did/do you do research?” The title of each bar represents the respondents’ answer to the question: “How much research have you undertaken to date?”***


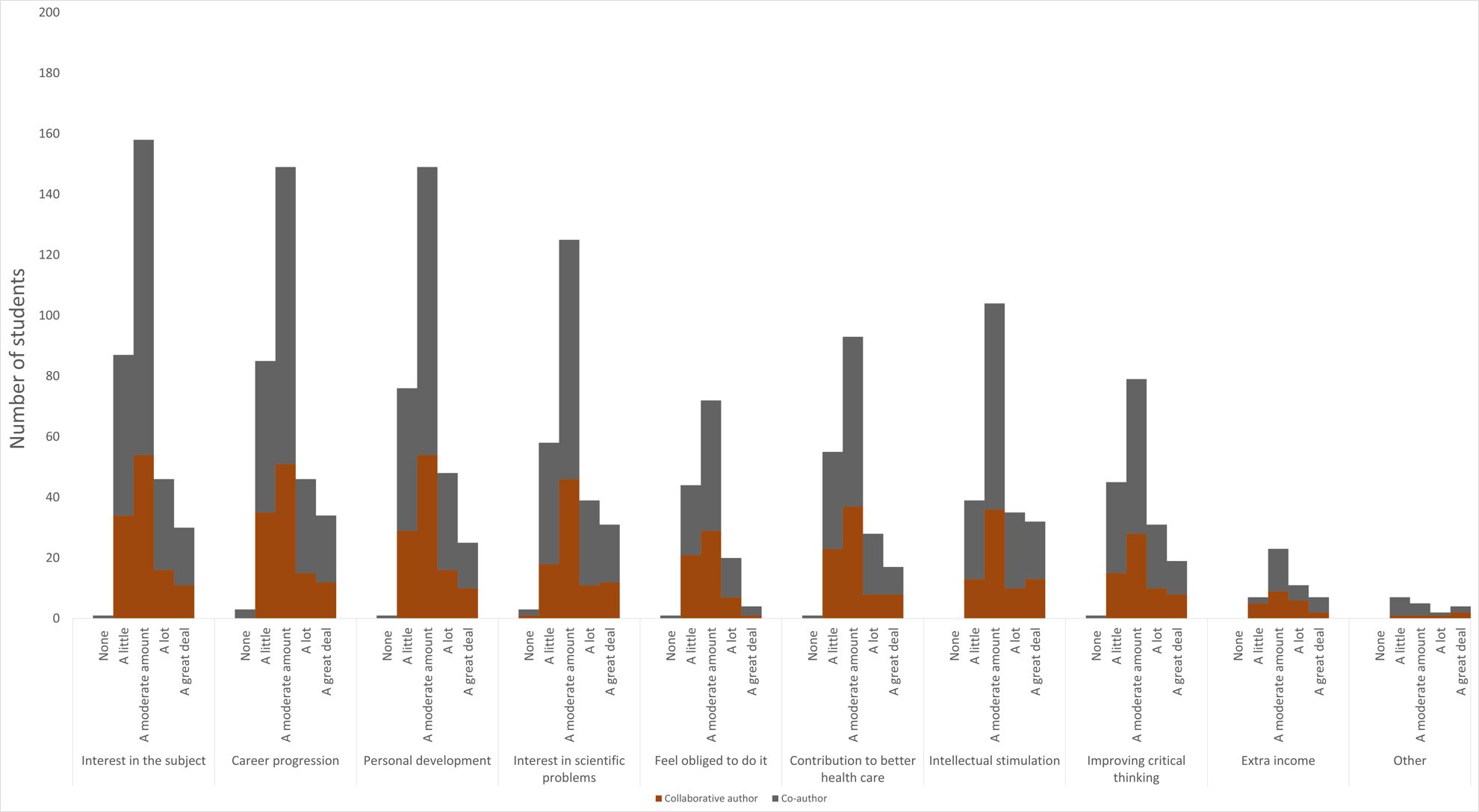

Supplement: Supplementary file 4 — Additional file 4: Appendix S4. Cluster bar graph of the number of students who have authored a publication by their motivation to do research. [file 12909_2023_4881_MOESM4_ESM.docx]
