# Supplementary material for: Cross-sectional Survey of Medical student perceptions of And desires for Research and Training pathways (SMART): an analysis of prospective cohort study of UK medical students
Source: BMC Med Educ. 2023 Dec 15;23:964. doi: 10.1186/s12909-023-04881-2 (PMC10725016; doi:10.1186/s12909-023-04881-2)
Supplement: Supplementary file 5 — Additional file 5: Appendix S5. Individual Responses to the Free Text Questions. [file 12909_2023_4881_MOESM5_ESM.docx]

Appendix S5 – Individual Responses to the Free Text Questions

| **Are there barriers preventing you from getting involved with research?** |
| --- |
| - Approaching doctors/surgeons in clinical practice to get involved  - Getting enough teaching on how to conduct research both from our university and the healthcare professionals involving you in their research  - Getting accredited for the amount of work you've put in research (I personally spent 6 weeks in a lab carrying out experiments and never got accredited for that and again in hospital collecting data for an audit and never got accredited)  - Finding people that would want to form a research group of similar experiences |
| - Difficulty in knowing 'how' to research/write papers  - No clear availability of supervisors  - Not knowing the utility of getting involved in research early on in medical school. A feeling that I found out 'too late'  - Juggling time & other commitments  - Very lengthy and dismissive process of submitting papers to journals and waiting months for feedback & unsure if they will publish. |
| - feel a stigma associated with medical students being involved with research compared to more senior members of staff  - limited opportunities for medical students to become involved  - many projects that do invite students tend to be ‘the grunt work’ with little hope of being published for your contributions |
| - Finding doctors willing to be supervisors   - Developing the statistical skills required   - Finding time for big audits |
| - Lack of time  - Not sure who to contact - Lack of resources |
| - Not having contacts or knowing how to network to get involved in research, particularly contacts in a specific area you might be interested in   - Not having time set aside during medical school to get involved in research projects clinicians are doing– if we want to get involved, that has to be in our own time as we are on placement 9-5 every weekday. This is challenging to manage, time-wise.  - Not knowing what ‘getting involved with research’ actually means, particularly as a medical student– my medical school (Leicester) has some brief teaching on quantitative research methods in the 1st year of the degree, but I found this wasn’t really to a sufficient level to actually know what you are doing when it comes to research (and also gave no acknowledgement for the importance of qualitative research!!!)- for me, that knowledge came from my taught masters degree which was created specifically to link research and clinical practice.   - I also was always concerned of being taken advantage of when senior doctors would speak about how great it would look for my CV if I joined their research project. It made me wonder whether they were looking to take advantage of my naïvety in the research world ie I would sift through the numerous research articles, collated data (despite not knowing what I was doing) etc so that they didn’t have to |
| - Not sure who to contact to get involved  - Not enough time during clinical years  - Feeling under confident from lack of experience |
| - school does not mention it nor provide education despite multiple instances prompting - was promised name on research paper by previous university 2x, both times did not happen, so issue with what is promised vs delivered. Students may do a lot of work to not receive any official thanks - time alongside medicalschool, in instances it is only holidays and breaks where there is time to conduct research where you may be expected for 12h days (in previous experience); often, research projects do not align with our timetable  - lack of opportunity. I have asked and it has been said to me that I must have prerequisit qualifications, or have finished medical school or there is nothing available in the near geographical region  - |
| - Social anxiety  - Inexperience  - Imposter syndrome  - Not having tons of experience in research already  - Opportunities shared by the medical school are not useful for specialities I would be interested in the future  - Other medical school commitments and load |
| - time - age - access - ID confirmation takes too long |
| - Unsure how to go about finding projects / how to get involved |
| * Not enough in the desired field  * difficult to get into, unless have contacts in that area. |
| 1) Not knowing who to approach for research opportunities  2) Not having enough teaching on how to carry out a research project/ present one |
| 1. Medical school does not provide opportunities or guide us in how to access these 2. I feel if you do not have the right connections, ie parents who are doctors or have friends who are doctors and are doing research, or you are greatly disadvantaged |
| 1. Time restraints - shorter summer holidays and during the year a lot of work for the medical degree  2. Where to find the opportunities can be hard to find |
| A good amount of external research projects are scheduled at an inconvenient time for full time students. |
| A lack of choice when it comes to selecting research projects. |
| A lack of opportunities from Leicester Medical School |
| A lack of opportunities, I was very lucky to have a Bmedsci built in my degree that enables me to get some research experience. But outside of this trying to get involved in research and reaching out/getting a response has been extremely challenging. Especially if you don't know someone or don't have a personal tutor with 'links'. |
| A lack of time since medical school studies take up a large portion of my time with free time being taken up by societies and extracurricular activities |
| A lot of assignments and coursework, placements, success and other works doesn't leave enough time |
| A lot of barriers exist such as few opportunities and not having enough information about how to do research |
| A lot of the time research is inaccesible- I.E if online there is never subtitles or interpreters |
| a lot to learn at medical school |
| absence contacts and absence of known resources |
| Absence of interest |
| Academic requirements which need attention and back to back placements which gives less time to participate and be a part of a research project |
| Access  Opportunities  Seems like a lot of hard work and very difficult to get a publication |
| Access is largely guided by what consultants you know, which ones are willing to give projects, respond to emails and how much they like you.     Also very dependant upon how much time you have to spare. The amount of work expected by consultants, for very little and not first authorship, is a great barrier to engagement and accessibility. |
| Access to clinicians willing to support students new to research   Removal of research from education points for FP allocation- reduced focus on doing this  Timing of when research is introduced during the medical degree |
| Access to funding and labs is limited. I sent 8 emails until someone took me in the lab and that was a virtual literature project. |
| Access to proper contact information and clear pathways of talking to the correct person. |
| Adds more years to degree, and I want to start earning asap |
| Affordability of attending university during the summer to engage in summer research projects. |
| Although I have got involved in research, I did find it difficult to find contacts/research projects that I could be involved in.   Many projects are not widely advertised and it's generally the same people who get the opportunity to do it (often they have friends or family who inform them about research opportunities). |
| Any knowledge we have regarding entry into research projects is what we find out for ourselves. We haven't been told the basics of how to seek opportunities, which is a daunting process to start when you've never had previous experience. |
| Aren’t told about where to look for projects that were interested in |
| As a graduate on an UG medical course I have to self fund. As I am from a low income household I have to work many hours a week to achieve this leaving me with little time for research. Paid research opportunities rarely cover the cost of the commute again making them unobtainable |
| As a medical student, I lack the time necessary to conduct research. Moreover, most research positions offered to medical students are unpaid, and I cannot afford to work for free. However, the ableism, amongst other forms of discrimination, and exploitation I have faced throughout all my past research experiences are the most substantial barriers preventing me from getting involved with research again. |
| As I am first gen into uni I have no knowledge of how research works, who to contact to get involved in research or how publication and peer review systems work. Uni gives no guidance and my peers are all able to get advice from parents who are doctors/have been to uni. |
| As I do not come from a financially well-off background and there are limited opportunities for financial help through University for me as an EU student the financial aspect is hard. If I want to do research I need to do it in my free time so there is no time left for a job for me. Furthermore, the scholarship on offer only provide me with the necessary financial support to cover my basic neeeds. |
| At the moment, the pay for Internships/summer projects is not much. This has put me off applying. It is less than typical UG demonstrator pay. |
| Availability of opportunities |
| Availability of staff and how many students they already have. Time constraints as a medical student if it’s not part of the curriculum. Lack of knowledge and skills about how to conduct research |
| Awareness and opportunity |
| Awareness of opportunities. Also, undergrads cannot volunteer in labs |
| balancing between different commitments is difficult |
| Barely any opportunities if any. |
| Barrier - don’t know where/ how to get involved. The knowledge of how to do this is not widespread. |
| Before intercalation, it seemed you had to have the right contacts in order to get involved with research in the pre-clinical and early clinical years of medicine. |
| Being dyslexic I find writinng difficult |
| Being female, time allowances |
| Being in first year so waiting to learn more of the course before getting involved with research |
| Busy schedule as a medical student so hard to fit in time consuming projects. Often not having the experience to know which projects would be promising in terms of learning or outcome (papers etc). No research mentorship, so had to do a lot of asking until I got involved with a project. |
| -Busy workload from core curriculum (it’s hard to find time)  -hard to find the right supervisor sometimes it’s just down t chance who you get to meet.  -can be very daunting when you don’t know where to start, it’s important to have the right support. I only really got this from doing an intercalated year which isnt available to all medical students. |
| Can’t afford it - placement don’t pay as much as working does |
| Can't be bothered |
| Can't think up a topic |
| commitments to medical school.  job to fund studying - less time |
| Competing useless tasks within medschool like portfolios or personal development plans that are neither high reward nor high yield. |
| Competition for funding; expenses for additional rent/living costs in wet-lab based summer projects. |
| Competition for limited spaces, COVID restrictions, lack of application guidance and support, difficulty in finding supervisors / mentors, limited time |
| Complete lack of experience  None from prior to uni and minimal from during this time. Intercalated year has provided the most experience but even that feels like it's meant to build upon experience we just don't have |
| Conferences are expensive and difficult to travel to now that they're becoming more in-person post-covid. The med-school has bursaries but there is a lot of admin to get one and you don't get the money until after the conference. |
| Confidence, opportunity and motivation |
| Consultants not willing to include medical students. |
| Contacting supervisors through email |
| Content and pacing of the course leaves little room to engage in extracurricular projects without it coming at the cost of academic performance.  If the structure of the course was shifted in years 2 and 3 so that SSC projects (at the very least) were given MORE time and students were given the opportunity to ACTUALLY do a proper research project with current research groups (as opposed to the internal fluff we’re expected to produce) then these modules would be so much more useful. |
| Content heavy nature of 1st year of graduate medicine particularly at Warwick, otherwise would’ve been interested in getting involved |
| Cost and time - need to work to fund degree |
| Cost of presenting research i.e. conferences   Accessibility and availabile time to complete extra committments on top of university requirements |
| Covid |
| covid - 2yrs ago |
| Covid   Difficult supervisors and technology |
| Covid closed the lab i was meant to be working in |
| Covid I suppose and the lack of information surrounding it |
| Covid meant we didn’t do our compulsory audit + QIP. |
| COVID pandemic |
| Covid pandemic made my research placement cancelled and I had to do online project. |
| COVID, living outwith the UK |
| Covid, pandemic, restrictions in the lab. |
| Covid-19 means there are fewer opportunities in research for students |
| Dealing with data is not that appealing to me. |
| Developing a close enough relationship with a senior colleague who would take on my ideas. Perhaps due to cultural differences |
| Different streams mean different people get the opportunities first |
| Difficult to engage with research and get involved in smaller towns. Also difficult with no contacts in the healthcare environment. People from (family) backgrounds with academia also tend to be better prepared to approach and get involved with research |
| Difficult to find doctors interested in working with students |
| Difficult to find opportunities |
| Difficult to find sufficient time around my studies and difficult to gain the relevant skills (e.g. on statistical analysis) |
| Difficult to get involved in research when you have no research experience - massive disadvantage for first generations in academia who have no contacts. |
| Difficult to know where to find opportunities |
| Difficult to know where to start, I’m quite shy as a person so wouldn’t really approach a random senior academic about getting involved. I’m not very flexible during the summer holidays as i have to earn money and that seems to be when many get their research done. |
| Difficult to know who to approach and what projects they have available. You also have a lot of emails that are not replied |
| Difficulty in contacting supervisors after projects have been completed to arrange publication |
| Difficulty in knowing how to approach people for research opportunities |
| difficulty in understanding how to get most information from article. |
| Difficulty making contacts |
| Difficulty of the subject  Heavy workload |
| Do not feel knowledgable enough on medical research to consider looking for opportunities, feel intimidated by the prospect. My first year medical research teaching was poor and disorganised, resulting in feeling confused about basic concepts. Now the teaching has improved, and i feel more secure in my understanding, but still I'm intimidated by the idea of taking part in research. |
| Does not appeal to me. |
| Doesn’t really interest me! |
| Doesn't peak my interest as much as other areas of medicine.  Seems to lack in the patient aspect of medicine |
| Don’t feel confident as don’t feel I have the appropriate knowledge or expertise about what research entails. |
| Don’t have time or money to dedicate to research outside of what is a compulsory part of the curriculum Not sure I can research anything useful at this stage/don’t know how to go about it |
| Don’t know a lot about it |
| Don’t know any other doctors (first doctor in the family) this makes it hard to network. Also, due to my background (ethnic minority + widening participation background) makes it harder to find a mentor with a similar background to me. Particularly I’m interested in surgery but hard to find other female surgeons in the fields I’m interested in |
| Don’t know how to get involved or how to make it legitimate research rather than something I’d do in school |
| Don’t know how.  Wasn’t told that I had to get involved.   Thought that I had to be established first. |
| Don’t know what’s out there. Unsure how to ask for opportunities |
| Don’t know where to go to get involved in research or what kind of research I can get involved in Haven’t been taught anything about audits and what they are |
| Don’t know where to look for information |
| Don’t know where to start |
| Don’t know where to start! Everyone always advertises that you can present posters to national conferences but what are posters? How do we do them? How do we get involved? |
| Don’t know where to start. Seems too daunting and time consuming. |
| Don’t know who to contact in order to get involved in projects |
| don’t know who to contact or how i can find out  Feel as though because i’m a student i won’t get it   Don’t know how qualified i am to take part in research |
| Don’t really know how to get involved in research or how to start a project. Also don’t really know much about how different studies are run. Additionally, we have a busy course and other things are prioritised. I don’t think my medical school really emphasises that medical students should get involved in actual research, I’ve only felt the need to get involved by speaking to other medical students with experience. |
| Don’t really know where to start and how to do it |
| Don’t really know where to start or who to contact. For audits, it’s been hard to get hold of ones that don’t last for over a year. Can be hard to get involved in existing ones, or getting in touch with those writing one, but again, it’s all about the right timing. Also am very overwhelmed with current state of where I stand in my degree and trying to balance out |
| Dont hear about the opportunities or know many ways of getting involved |
| Don't know about it |
| Don't know enough about available opportunities to get involved in  Wouldn't know what to do or who to talk to if I had an idea that I wanted to pursue |
| Dont know how |
| don't know how to |
| Don't know how to access research opportunities/how to contact researchers etc. |
| don't know how to begin |
| don't know how to find roles that are suitable for medical students |
| Don't know how to start or who to contact if interested |
| Don't know I'm only a 1st year |
| don't know where to find a research project as a clinical student;   my mental health |
| dont know where to get started |
| Don't know who to approach  Unsure of methods to start research project |
| Don't know who to contact No prior guided experience to do research project |
| Don't really know how to get involved in writing papers rather than data collection |
| Don't really know much about research and how it works or who to contact to get involved |
| Don't really know where to start, worried about the time commitment on top of medical school. |
| Don't really know who to speak to about research and how I can get involved |
| Early in medical school, no and little network, little knowledge on what research is |
| English being my 3rd language |
| Entirely online learning makes networking with academics very difficult |
| Exposure to research and how to approach writing papers  How to approach clinicians to get more experience in research |
| Exposure, opportunities to get involved, uncertainty regarding how to get into research |
| Fear of being unsupported while undertaking research especially with little to no prior experience    Doubts about my own time management skills - having to juggle research, studies, extracurricular activities, personal life |
| Feel completely out of my depth in the research field |
| Few places and loads of competitions  No financial support so have a part time job in summer  Have to work as a carer  No confidence in my abilities |
| Financial issues and paying back student loans. |
| Financial support sometimes |
| Find it difficult to know how to connect with supervisors. |
| Finding opportunities and contacts to get involved in research projects |
| Finding people to help |
| Finding projects |
| Finding research that is the right fit. Often you’ll find opportunities by meeting clinicians, but these aren’t necessarily in areas of particular interest |
| Finding someone to write a reference letter |
| Finding supervisors who are engaged in taking on students and supporting them with research. |
| Finding supportive mentors |
| Finding the means to participate in research (difficulty in networking) and trying to find time for research in between studies |
| Finding the proper professor and where to start. |
| finding the right people to join with and finding something that interests you |
| Finding the time alongside an intense course |
| Finding time alongside the rest of the course content to pursue research can be tricky |
| Finding time to do research alongside studying for medical exams is difficult. |
| Finding time to get involved with research, not knowing people working in fields I find interesting and would want to get involved in. Research seems a bit like it's the people you know that is important as opposed to what you know. |
| Fitting it into medical school commitments |
| Focus of AFPs on deciles etc - not taking into account factors that may have contributed to less favourable performance e.g. disability or mental health problem |
| for undergraduate students with no previous experience in research there are few ways in which to get involved with beneficial opportunities |
| From my experience, research that are not compulsory (i.e. not part of the school curriculum) are very competitive. Only a select few are able to gain a place in research. For example, there is an opportunity for medical students to partake in MB-PhD programme. However, because I am an international student, according to the webminar I attended, the option will only be open to me if I were to rank number 1 in my cohort. Additionally, the only research opportunity open to first-year medical students is the Laidlaw scholarship program, which is quite competitive. Being a research assistant is also not an option. According to my university, medicine is too heavy a course for us to volunteer as a research assistant.  My experience may not and probably is not representative of everyone's. I hope the area of research will be more accessible to me as I progress in my studies. |
| From my experience, there does not appear to be a shortage of available projects. However, it often seems to be a case of 'not what you know but who you know', and the luck of the environment that you are in to find suitable research projects. There does not seem to be any wide reaching schemes that are available to help anyone (regardless of social background!) keen to develop their research 'skills'. |
| funding |
| Funding is difficult to find as a student, especially in the field of neuroscience.  Furthermore many great labs are not keen on taking on medical students |
| Funding. Time. |
| Getting involved in research depends on exposure anand good guidance and supervision which depends on the research department. In my experience this can be varied. There is also a lot of work and learning involved especially for medical students doing it for the first time and this can be even more difficult if no formal teaching or supervision is inadequate. Sometimes timing of projects is too short (4-6 weeks) and balancing research activities with medical school learning may also be a factor which discourages some students. Really have to be highly organised and motivated to pursue both. However, it is very worthwhile and lots of skills can be developed. |
| Getting opportunities to be involved |
| Guidance from the university is a barrier, however I’ve been able to get involved with it through contacts I’ve made during my clinical placements. One reason why I’ve tried to get involved with it during my studies is because it helps with future jobs (currently). I think if medical students were told about the benefits of research in terms of career prospects then more would engage with it. |
| Hard to find opportunities that align with interests. Seems like it's more who you know rather than what you know. |
| Hard to find opportunities. |
| Hard to find projects whilst at clinical school, no protected time for research |
| Hard to know how to begin |
| Hard to know where to get started and who to contact/ what can do with little experience |
| Have 0 contacts in the medical profession. No family or family friends who are doctors. So much nepotism goes on with medical research authorships. |
| Have asked people in hospitals but they don’t get back to me often |
| Have been told by university tutors that students lack experience and the only way to engage in research is to do additional degrees e.g. intercalation. |
| Haven’t really been introduced to medical research and need to look into it myself. |
| Haven't had the teaching or opportunities to get involved yet |
| Having a mentor to help guide you with how is best to get these opportunities |
| Having no previous experience in research or learning about it in school has been a barrier when applying to research programmes. |
| Having teaching earlier on on how to get involved in research would be useful.     Greater publication of opportunity’s is needed as many research roles are only shared through word of mouth or knowing certain people |
| Having the time! Suitable opportunities available within research teams, receiving replies when contacting research times |
| Heavy commitments and less time to manage |
| Heavy workload, limited opportunities (20 summer studentships for 200 students) |
| High competition |
| High competition |
| Home situation and sporting commitments make it difficult to leave home to involve myself in research posts - but these are mostly my own choices, so I could change this if I gave up certain other things I do outside of my studies. |
| How to contact the lab |
| how to go about research? more education on where to get started and WHEN best to do it? how early in medical school to start? |
| I am a mature student with children, so I have less time to engage in extracurricular research now than I did when I was first an undergraduate. I do not consider this an absolute barrier, it merely makes it slightly less easy than it could be. For the most part, I have many opportunities and few restrictions. |
| I am an international student, and many research opportunities are only available to UK nationals or those with an ILR (Indefinite Leave To Remain). |
| I am not sure on what opportunities are out there, I am not sure what is involved in undertaking research.   I do not have the basic research knowledge like how to do a literature search or undertake a systematic review.  I know nothing about statistics. |
| I am struggling to find any opportunities available to me, also having the time around my studies. |
| I am struggling to get involved in research. I have tried contacting numerous people but never get a response back. The whole process needs demystifying for me. |
| I am unable who to contact, and if my level of education would even qualify me to be a researcher |
| I am unaware of the different research opportunities available. |
| I am unsure how to go about getting started. |
| I am unsure on how to access supervisors and get started. |
| I am unsure where to start, how to carry out the research/find topics to research, how to do the statistics/what would be the outcome of the research. I find statistics difficult and this deters me from getting involved. We were never taught what an audit was or how to get involved in one. |
| I am usually extremely confused on where to start and what to do, more teaching on how exactly we could start a research project would be very helpful |
| I believe sometimes it's due to luck, as a couple of projects I got involved in have not lead to any presentation-publication. So it's not really a barrier, but more so a problem of not finding the people that would mentor students through the process |
| I can’t find any protects to get involved with even tho I really want to |
| I can't afford to take part in research, most opportunities are unpaid however I need to spend every spare minute I have to fund medical school by working different jobs. This also leaves me with little to no free time to take part in research. Also I do not know how to get involved, my medical school does not inform us of opportunities or give support regarding any external applications. I feel at a disadvantage when applying for the limited paid opportunities as I have no research experience and I have no clue what to write on an application form. My family can't help, one of my parents is deceased and the other can't help with applications as English is not their first language. I have no other medical contacts either or any other help. |
| I cant find the help to start and I don’t know where to begin. It is daunting to start by myself |
| I do not enjoy research |
| I do not feel confident to be able to |
| I do not have many opportunities or connections to people in research or academia |
| I do not know what steps I need to take if I want to go into research. I also do not know at which point in my career would be the best time to do so |
| I do not know where to start |
| I do not know where to start. Who to reach out. I haven’t found research I would be interested in. I tired to take part in one research as a data collector but due to lack of communication and weak leadership, I could not complete it because i didn’t know what i was supposed to do. I reached out to many people but only got vague answers |
| I don’t enjoy it |
| I don’t feel clever enough |
| I don’t feel like I have time while at university, and also lack connections to projects etc |
| I don’t have the time and don’t have the interest |
| I don’t know enough about it and I haven’t found a field in which I am interested enough to do research on |
| I don’t know how to get involved |
| I don’t know how to get involved |
| I don’t know how to get involved and get authorship |
| I don’t know how to write essays, how to approach research topics, how to carry out research methodology. |
| I don’t know what research I should be doing or how to get into it because this isn’t taught in medical school |
| I don’t know where to go or what to do to learn about research or research opportunities. I also live and studied in school in an area of socioeconomic deprivation. |
| I don’t know where to look for research opportunities. I also don’t know if I would be able to get involved in anything due to only being a second year. |
| I don’t know where to start |
| I don’t know where to start or how important it is. I feel like if I give too much time to it it could affect my studies |
| I don’t know where to start I’m afraid if I ask for guidance or for a project I’ll get turned down |
| I don’t know who to reach out to if I am interested in taking part. I am also worried that supervisors find it annoying when I contact them. |
| I don’t really know about the opportunities or have any of the relevant skills yet, we do a research project in 4th year but it isn’t really mentioned until then. |
| I don’t really know how to get involved. Also, with the workload of medical school I don’t feel I have the time to. |
| I don’t really know how to  No family or anyone to guide me  Never really had any guidance from uni   Lack of contacts |
| I don’t really know where I would start or how to start researching, also as a student any area of interest I may have I feel like I don’t know enough to do my own research |
| I don’t think I’ve been equipped to participate in research and therefore I’m not confident to do so. How to get involved in research as well isn’t particularly well advertised as a medical student |
| I don’t think my research would be good enough  I’m unsure what opportunities are available to me |
| I don’t want to |
| I don't enjoy research |
| I dont feel like I have the opportunity to be involved in research. Covid and lack of time and funding has significantly reduced chances of involvement |
| I don't have a good CV to email and ask for research opportunities  I don't know what type of research I can do apart from my SSC |
| I don't know how and what it entails. |
| I dont know how to |
| I don't know how to access the different research teams and have had no training about it. |
| I don't know how to get involved as a student |
| I don't know how to get involved or find opportunities to get involved |
| I don't know how to start. How do I get to be part of research. Even if I was part of a research project, I do not know the proper way of undertaking it. What purpose does a research project at my level of academia have? |
| I don't know the pathways into research and the different ways to get involved. |
| I dont know where to start or how to get involved |
| I don't know where to start to be honest and ask the professor for research. |
| I don't really know about opportunities and don't have much extra time, also I don't understand statistics |
| I don't really know about the opportunities out there. I don't know what I'm interested in. I don't have a lot of background information about what can be done by medical students so even if i signed up for something I would have no idea what i was doing |
| I don't really know what opportunities I would be about to participate in based on my current skill set |
| I dont think I have the tools to conduct a fair research project. If I was taught better I believe there is so much more research projects I would do. |
| I don't think many opportunities get presented to us as medical students, especially for research. At least in Lancaster, the opportunity to do research does not come often. Furthermore, I think there is a barrier to research in that often people with already more experience are likely to get selected to take part rather than people with little-to-no experience. |
| I don't want to |
| I feel am time limited due to studying medicine and attending my placements |
| I feel as though i do not have enough time alongside medicine to get involved with research |
| I feel as though others who have connections have easier opportunities to take part in research whereas I do not know anyone in the medical field who I could work with and only come across opportunities during placement (which is a very short time period too) |
| I feel I do not have enough basic level knowledge to take part in academia or research |
| I feel like I am not qualified enough because of the lack of experience and I am not well informed with how research works. Also there is a fear that I am using valuable time on research when I can use that time to study or even take that time to relax. It would still be possible for me to do research only if I feel like I am qualified and I know what I must do. |
| I feel like I don’t know enough about it or how to get involved - may not be enough support or guidance |
| I feel my medical school does not offer opportunities to get involved in research as an undergraduate.   I have tried to set up my own link with a local GP practice to conduct an audit but due to covid this has not been possible |
| I feel that I don’t know a lot about it. As no one in my family has been to university, I have no one close to help me out with this, my peers who have academics and healthcare professionals in their family are at an advantage in this respect |
| I feel that the medical course is already demanding enough, so there is little time to get involved in research in a meaningful way without sacrificing some aspects of your studies. There is no time set aside within the curriculum for such endeavours. |
| I feel that there have not been many opportunities for me to be involved in research while in medical school as the opportunities are few and I wasn't one of the chosen ones. Not only that, \I have tried asking a couple of consultants however, the consultants I've asked were not involved in research. There also isn't sufficient time set aside in medical school to do a block of research. Lastly, I chose not to intercalate as I would have to fork out extra money |
| I feel there is a lot of barriers in terms of who you know. Many people who get published are through contacts. |
| I find it difficult to find research opportunities. |
| I find it difficult to find research projects to get involved in |
| I found it difficult as whenever I approached individuals about getting involved in research as it usually ended up with myself having to do the entire project with little support or know how. We were not thought how to go about publishing. In addition, our university does not allow/have an account for major publishing companies and therefore the full text is unavailable to be read for free, meaning that my published work will not be read. |
| I had to fund my medical degree as this was a second degree, therefore I had to prioritise working for tuition fees in spare time instead of getting involved in research.     Sometimes, it is who you know for getting involved in research.     Often, when I have asked supervisors that I would be keen to do research - this has been ignored, I assume they were not interested in research or too busy to signpost to relevant colleagues. |
| I have children and it's challenging to have a work life balance while studying medicine |
| I have no family in academia or medicine, and there seems to be many 'unwritten rules' about how to secure publications. It seems there's easier access to my peers who have 'connections' within medicine. Medical school have taught us how to read research and to, an extent, how to conduct research. However, without these connections it's difficult to have opportunities to complete this. |
| I have no idea how to get involved in research. My medical school provides no guidance on how to conduct research projects, make posters, etc. I feel wholly unprepared and therefore would be hesitant to approach any healthcare professionals about doing research |
| I have no idea how to get involved. When I have been involved in research previously, the co-authors went back on their word and didn't put my name on the papers (this has happened twice), so have become very unmotivated to try again. |
| I have no idea what research entails- so I’d lack of knowledge on the process, lack of resources shown to me to educate on the process and a further lack of connections (network) to undertake or even learn again about the process of research |
| I have seen people go through the process of research and it has put me off. |
| I haven't tried myself yet to get involved in research but my upper year buddy group told me it's quite difficult to get a research opportunity |
| I just don’t even know where to start |
| I just feel as research is not advertised. We are not really given the opportunities to do it. There are always adverts for poster presentations at conferences and such but no opportunity to actually conduct the research to present at these. |
| I live at home with my parents and commute into university due to my household income and not being able to afford to live out. I don't have as much time to get involved with projects since I'm commuting for longer times or I'm working a paid job. This makes it more difficult to dedicate time to unpaid research that might not always be fruitful |
| I think I wasn’t aware of how time consuming it can be and the level of commitment needed |
| I think it is a personal lack of experience with how the process works. Coming from a non-science background there is a large portion of awareness on how getting into research works. I think that potentially there could be more targeted teaching on a graduate entry course which actively encourages people from non-science backgrounds to apply |
| I think it is very difficult to know how to get involved with research. Many opportunities are found coincidentally and as a result of being in the right place at the right time. There is no system to help students involved with research. Many students feel inadequately prepared to get involved with research although they want to and know that it is important for their future career. |
| I think it’s because I’m in first year so I’m not sure of what opportunities are available for research. |
| I think lecturers can use more evidence from recent publications to consolidate our knowledge. More research opportunities should be available to medical students. COVID has disrupted a lot of things but more places should open to student and they should be encouraged to publish. |
| I think the barrier when comparing to other medical schools is that a lot of opportunities are not advertised to us. As medical students we are in a privileged position to be exposed to so much and I have seen many peers from other schools take up opportunities to travel for conferences and publish earlier on in medical school. Unfortunately, from my experience I have seen that we as students have to take the extra mile to find these opportunities. |
| I think the main barriers would be a lack of contacts as an undergraduate and a lack of extra free time during medical studies |
| I think you need to network with people and make contacts to gain opportunities, which I haven’t done |
| I want to get involved in research but there are several barriers. I have tried to get involved in the Cascade project by STARTSurg but my university didn't allowed it. Then I have been trying to get research experience thorough courses, webinars, internships,... It is not much going on and it is confusing. I have managed to apply to two clinical research internships to gain some experience, currently I am waiting the results.  I believe people doesn't really get involved in research because you need to be extremely proactive and keep trying. |
| I wanted to do academic foundation post but my EPM wasn’t high enough to be even considered for any of these. Waste of time writing the application. |
| I was ill and my grades went down resulting in being in low decile and I can't really get into competition to get research |
| I worry a lot about balancing my time between studies and research, that if I commit to research will it be at a detriment to my studies? |
| I would like to be more involved but I think early on in the course it is hard to know what opportunities are available to you. |
| I would love to do a phD but that requires doing 10 years at university and although you get paid to do one, it does not start your way up the career ladder and I have to make some money to support my family |
| I wouldn’t feel “clever enough” and also worry about being involved alongside my academics as it isn’t clearly shown possible by others |
| I’m not sure how to get involved. |
| I’ve struggled to have access to research projects and I don’t really know what to do. I’m hoping to do more next year |
| I'm exhausted. I just want to be able to relax and be a normal person and not have to juggle a million and one things to be perceived as keeping up with everyone else. Also, how do you even get involved in research? I have no lab skills, I don't have much to add to a research team, and I don't have any links to anyone doing research. Plus, a lot of it going on just doesn't seem interesting to me |
| I'm in Year 1 so there are not many opportunities for research |
| I'm not even aware of what I can research, who can help me with it at university, or if my ideas are even worth researching about despite looking up on Google. |
| I'm not sure if this counts as a barrier, but there isn't much opportunities for research in the earlier years especially first year. I suppose it could also be due to our lack of clinical knowledge |
| In preclinical years difficult to find mentors. Many mentors seem to want students with prior research experience, so to gain research experience you need prior experience. |
| In preclinical years, many projects took place over summer when I wouldn’t be able to stay in London (only time I can visit family who live abroad etc).  In 3rd year during my iBSc I got my first taste of research and really enjoyed it.  Now in clinical years, I think it’s difficult to balance placement, revision and a project - I will be attempting it but I’m not sure how doable it will be!  Generally, I’ve also found it quite tricky to get access to projects in topics I’m interested in - a lot of people involved seem to have personal connections who help them get a foot in the door. |
| In the earlier years of medical school, it was a lack of knowledge on how to perform and get involved in research. I didn’t think it was something I was able to do as I didn’t know anything about it. Later on, I’ve found with some consultants (not all) that they don’t take me as seriously as they do my male colleagues, and are more likely to give opportunities and encouragement to my male colleagues than to me. |
| inability finding opportunities which I am eligible for and which I have been able to get onto |
| Inability to find mentors or professionals who are willing to supervise and guide |
| Inadequate information on research opporunities available, as well as on how to get involved (networking, finding mentors etc.) |
| Inadequate teaching / guidance from the medical school |
| Inadequate teaching of how to get involved and how to get published |
| Insufficient opportunities, not understanding process of research |
| interest in neurosurgical research is difficult in a city without neurosurgery |
| Introduction to the complexities of research is quite daunting. Writing up a research protocol was compulsory as part of our year assessment, however a lot of teaching was vague and hard to apply to a very specific research question that each student had (as by nature, individual research protocols are vastly different from each other). |
| It can be difficult to know where to look to get involved, we are given very little guidance. There are many types of research projects that you can do, but we are not advertised these or given guidance to how we can go about it. Also, a lot of the time I feel I am very busy balancing studying and family/social events as well as extra-curriculars. If I were to take on a project, I believe I would be over stretched or not be able to give 100%. |
| it can be hard to find good supervisors or opportunities that lead to publication |
| It is difficult for medical students to find clinicians who want to help them with research and also difficult to find time |
| It is hard to access information about research and I’m not sure how to get involved in research. |
| It is hard to find research positions due to limited availability. |
| It is hard to know how exactly to go about contacting the right people or asking the right questions to get involved. |
| It is so hard for students to get involved in research, it is either not accessibly promoted or just requires an immense amount of emailing around. Additionally, the way in which students are allowed to be involved with research limits their understanding of the research process as a whole. Preventing them to have the transferable skills when they are managing their own research project in the future. |
| It seems very competitive |
| It takes many years to be involved in research and some of us need to earn money as soon as possible so it does not seem feasible. |
| It tends to be who you know whether or not it is offered to you. I have friends who have relatives in medicine who have offered for them to help in research. Students who do not have these connections have to seek out opportunities and it’s never been told to us how is best to do this. We are told to undertake audits in fourth year but never even told how or even what these are. The only reason I now know what these are is because a kind pharmacist explained it to me one day when I was just sitting about the ward. As someone who suffers with anxiety in social situations it can be difficult to seek out these opportunities. |
| It’s difficult as you need contacts and most people you reach out to don’t respond |
| It’s hard to find suitable supervisors for a project and then to find time to actually do the research while also doing a medical degree |
| It’s not a huge part of the course. |
| It’s not as interesting as clinical medicine |
| It’s not clear where to start when trying to get involved in research |
| It’s often who know as to what research you can take part in (who will supervise you). A big barrier is also time. Why would I complete research for free when I could be working part-time to gain an income whilst at university? |
| It’s so cliquey, once you get in it’s fine but it’s a lot of work to even be considered. As well a lot of students ONLY do research for points which gives the rest of us a bad rap as they aren’t always the best at performing the research |
| it's difficult to get consultants etc to reply in order to get involved in the first place plus we don't get much support in terms of publishing projects |
| It's hard to find projects |
| It's not really something that's encouraged, I wouldn't know where to start or how to get into it |
| It's not very well explained at Leeds. No one clearly explains how the researchers project works, why abstracts and oral presentation/ conferences are important.   There's not that many clear opportunities to get involved. It's very much who you know.  There's high barriers to entry |
| I've done some research in my previous degree and haven't done much research this year except SSC. Summer studentship is available but I heard only 20 places are there for the whole year of 300 students |
| Joggling between coursework and research.  Applied for a few positions to do research but there was no funding and have to work in the summer to earn some cash. |
| Just not knowing how to go about initiating it |
| Just the general workload of medical school? It’s really hard to find time to do it aside from our holidays |
| Keeping up with academic requirements |
| Knowing how to apply for research projects and where to look to find them |
| Knowing how to start |
| Knowing researchers to work with-- have to email tutors asking whether they are doing any researchers rather than having family/ family friends who are looking for students to help them on medical research projects |
| Knowing where to find all the research opportunities    Actually being interested in research |
| knowing where to find opportunities to get involved in research, time constraints around an already demanding degree |
| Knowing who to contact and having the time to do it. |
| Knowing who to talk to about it |
| Knowledge and opportunities |
| Knowledge of what is happening, time, funding |
| Knowledge, guidance, teaching |
| Lack of access |
| Lack of access to clinical settings |
| Lack of access to contacts or information on how to get involved with medical research.  Reluctance of medical professionals to allow participation in research due to lack of previous experience. |
| Lack of access to research opportunities |
| Lack of accessibility   The feeling that you need to already have good connections such as family/family friends to be able to be involved in such research, and for open applications they’re heavily oversubscribed by people like myself |
| Lack of advertisement to students to get involved with these kind of opportunities |
| lack of advice  lack of supervisors  lack of teaching about research |
| Lack of awareness |
| Lack of awareness of available opportunities as these are not advertised widely. Also a lot of research opportunities require prior research experience which makes it difficult to gain a place in a research project as a pre-clinical medical student. |
| Lack of awareness of opportunities |
| Lack of connections |
| Lack of connections that other students from privileged backgrounds have (eg due to family)  Lack of time outside of studies to participate in research   Not knowing where to find opportunities |
| Lack of connections. Lack of knowledge about opportunity. Lack of understanding of what it would entail. |
| Lack of contact points in lower years |
| Lack of contacts in the medical world.  Lack of knowledge on where to even start, who to contact, how to do the work even if I was involved.  Competition with other med students all trying to apply for very limited research opportunities. |
| Lack of contacts, knowledge about how to get involved |
| Lack of contacts. This may be due to social barriers (e.g. Anxiety or lack of family members in the medical field to help). |
| Lack of contacts/networks to be able to get involved. Not knowing who to go to/how to get involved as this isn't explicitly told. |
| Lack of direction and informations from the medical school to start research |
| Lack of experience + knowledge in research methods  Lack of networks / opportunity   Intimidating |
| Lack of experience and lack of knowledge about available opportunities and how to pursue them |
| Lack of experience and opportunities (when in first year at medicsl school) |
| Lack of experience/low confidence in my abilities to conduct and publish research |
| Lack of exposure |
| lack of familiarity with research and how to start, lack of enough training at uni to do proper research |
| Lack of guidance and availability of research projects |
| Lack of guidance and support regarding the process of publishing and how to access research. |
| Lack of ideas |
| Lack of incentive, lack of prospects, lack of interest, lab environment not appealing |
| lack of info |
| Lack of information - and what little there is, is not presented in a way to make it seem interesting |
| Lack of information about how to get into research, I.e. who to contact, how to get selected, how to write a covering letter/CV to be accepted. Also how to find opportunities for research. |
| Lack of information about opportunities |
| Lack of information about ways to get involved. Lack of interest from researchers/lecturers/cliniciand at university to involve students. |
| Lack of information on how to get into it   The topics I am most interested, mostly pertaining to race, ethnicity, intersectionality etc, are deemed taboo topics by most white academics who take up the majority of the field so it’s not easy to do what I am interested in |
| Lack of information on how to get involved. |
| Lack of information on what is needed/how to go about finding research projects. Competitiveness of applying for academic foundation programmes. |
| Lack of information  Don't know where to start |
| Lack of know how and support academically and financially |
| Lack of knowledge |
| Lack of knowledge about additional opportunities to get involved with research |
| Lack of knowledge about how to get into research. Lack of contacts. Lack of knowledge about importance of research. |
| Lack of knowledge about how to start and lack of time |
| lack of knowledge about opportunites |
| Lack of knowledge about research |
| Lack of knowledge about research. How to get into it, how to do it, what are the resources that I need to use. |
| Lack of knowledge about what research I can do  Very little time |
| Lack of knowledge about who to contact, and lack of knowledge about what it entails  Lack of time |
| Lack of knowledge and contacts |
| Lack of knowledge and direction |
| Lack of knowledge and experience as well as time management problems. |
| Lack of knowledge and network/opportunities |
| lack of knowledge of how to conduct research  lack of contacts |
| Lack of knowledge of how to enter research |
| lack of knowledge of how to get into research |
| Lack of knowledge of opportunities |
| Lack of knowledge of opportunities |
| Lack of knowledge of the approach and process of beginning or getting involved with research opportunities in the first place, and with knowing how to actually undertake the research.    lack of confidence in research abilities    Lack of time due to other university/study commitments and demands |
| Lack of knowledge on getting into research and future career pathways |
| Lack of knowledge on how best to integrate this with medical school commitments. |
| Lack of knowledge on how to start/approach this  No teaching |
| lack of knowledge on where to start! |
| Lack of knowledge on where to start, who to talk to.  Lack of confidence regarding research |
| Lack of knowledge. |
| Lack of knowledge. Medical research is very advanced. I previously tried to get involved in research but found I didn't have enough medical knowledge yet to properly follow the papers I was reading.  We are also given no guidance on how or who to approach if we are interested in getting involved in research. I was supported once I reached out to a couple of my lecturers for advice, but still felt I was embarrassing myself and not going about trying to get involved in research the right way. |
| Lack of known contacts  Time constraints - juggling the medical degree, research and other extracurricular activities |
| Lack of mentors. You have to get lucky and find someone who publishes prolifically to take you under their wing, harder to do if you're more introverted! |
| Lack of networking opportunities |
| lack of opportunites |
| Lack of opportunities |
| Lack of opportunities |
| Lack of opportunities |
| Lack of opportunities and requirement to secure funding to gain research experience. |
| Lack of opportunities- especially during COVID/lack of placement |
| Lack of opportunities in lower years.   Less importance on research during curriculum, had to scout opportunities if you knew who to ask but wasn’t as accessible to everyone |
| lack of opportunities in uni / lack of info from uni |
| Lack of opportunities provided by the university |
| Lack of opportunities  Lack of free time as I work whilst studying |
| Lack of opportunities, very much a case of knowing the right person or being in the right place at the right time in order to get involved. Also I am not planning to apply for a competitive specialty so there is less pressure to get publications. |
| Lack of opportunity |
| Lack of opportunity  Lack of knowledge/ guidance |
| Lack of opportunity / time to find projects |
| lack of oppotunity |
| Lack of Personal time and availability of doctors to help with research |
| lack of possibility to get involved |
| Lack of project availability at my university  Unexplained protocol/difficulty in contacting potential supervisors   Lack of experience + lack of opportunity given  Difficulty in selecting supervisors that match career and research interests |
| Lack of support and guidance. Supervisors that don’t have time to meet with you and expect you to work out how to do things (grant applications etc) by yourself. |
| Lack of teaching, lack of support, have to pay to access a significant number of articles, have to pay to attend conferences and publish |
| Lack of time |
| Lack of time |
| Lack of time |
| Lack of time |
| lack of time and also not having a supportive supervisor |
| Lack of time and dedicated supervisor |
| Lack of time and feeling intimidated or like an imposter |
| Lack of time and knowledge on how to do individual research products/ apply/ get funding etc.. |
| Lack of time and opportunities to engage with research |
| Lack of time as a medical student is a massive barrier.  Knowing who to contact or what to do to start or get involved with a research project is difficult. |
| Lack of time during clinical years. Not being given any guidance on how to get involved in research. |
| Lack of time or tutors who are looking for students to assist with/ students to support with research |
| Lack of time to balance degree with research project. A lot of time in Ward days is wasted when it could be used for this |
| Lack of time to find and/ or conduct research |
| Lack of time to get involved with research alongside studying |
| lack of time  low amount of projects available that have high probability that one will end up having a substantial role |
| Lack of time, lack of awareness of what I could do, lack of confidence in knowing what to do therefore not taking available opportunities |
| Lack of understanding   Lack of roles available |
| lack of understanding about how to get research positions |
| Lack of understanding about the intricacies of research and lack of appealing incentives. |
| Lack of understanding and teaching   Lack of mentors  Insufficient time allocated to research on the curriculum |
| Lack of understanding of how to get involved in these opportunities. |
| learning/knowing about opportunities  funding availability   stage of degree, experience/expertise required eg. coding ability/clinical projects |
| Less opportunities to take part |
| Limitations of time, limited willingness to spend time in research |
| Limited opportunities   Wouldn’t know how |
| Limited opportunities, especially without much experience of my own. I find that while most scholarships etc advertise as not requiring experience, it's very often the post-grads who have already published papers getting the places. While I understand why, I would like to see more scholarships reserved for those of us who have not been able to get involved in research before. |
| Limited time - busy with studying |
| Limited time (working in free time where possible) to pursuing opportunities apart from elements included within course |
| limited understanding and knowledge about how to get involved |
| Little guidance on how to start / the process of running or being involved in a research project. In terms of getting research recognised, the university encourages us to do projects as part of our Student Selected Components but doesn’t give guidance on how to take this further and present it or get it published |
| Little knowledge |
| Little time and opportunity to get involved |
| Little to no opportunities |
| Little understanding on how to go about completing research, time, types of research, benefits of research etc. |
| LMS is not very research focused do not give much assistance with publications should people want to pursuit them. There teaching on writing academic literature I would say is quite poor compared to my intercalating uni |
| Loads really, I was lucky in that my professional mentor Dr Monaghan, who I was assigned to at the end of second year, Sat down with me and had a conversation with me about what I wanted to do and I mentioned research - before then I’d done nothing and, despite having an original idea that’s now going to be published as a paper in a high quality journal, I had no idea how to even begin to start in research. Since then I’ve gone through an ethics board, helped in an audit and case report and written a paper, and it’s not an exaggeration to say that had I not had such an encouraging professional mentor I wouldn’t have done any of that and would have had no idea where to start. Student choice projects are good for getting involved in interesting research, but it definitely feels like the ‘how to’ of getting involved isn’t explained at all in medical school, which is a shame because most people I’ve spoken to have expressed an interest without knowing how to start (and indeed, those that do start seem to go on to do loads). To give a medical school example, opposite the main teaching hospital in Bristol there’s an education centre which has loads of different specialities doing different research - the fact I only found out about this trough my mentor was truly surprising to me - if I were the Med school I’d be scheduling time for students to look around and get interested in this hub of research! The importance of research is stressed to us, yet getting involved requires a lot of work that could be made easier by the Med school.   One final note - from speaking to many other students, ethics committees are by far the worst part of every paper writing process. On a personal level I was delayed by 3 months, and while that’s obviously an exceptional circumstance, there should definitely be a students ‘how to’ guide or a clear point of contact for students interested so they don’t get put off at the first hurdle. |
| Location and opportunity to connect with professionals in the area of interest  Lack of training on research skills  Lack of compensation for time |
| Low pay in academia relative to other career paths |
| Main barrier is time - difficult to find an extra 10 hours a week which certain projects have required from me to get involved. This especially affects poorer students as many of us work part-time on top of the degree.    Other barrier is contacts - sometimes its harder to find a supervisor happy to take you on, and also can be hard to find the contact details of academics for you to contact. |
| mainly time |
| Managing time for academia and job as well as research which isn’t compulsory at the moment meaning having to make additional time for it which is difficult. |
| Many HYMS tutors do not themselves get involved in research so it can be very hard to find people to conduct research with. |
| Mental health disabilities, lack of time due to compulsory course components |
| Money and time constraints |
| Money for publication Money for travel for presentations |
| Money required to travel to conferences.   As a graduate student I need to work to find my studies due to reduced maintenance loan so don’t have time to take on lots of extra research roles in my free time. |
| Money to do an intercalated/further degree |
| Money, time, contacts,... |
| More published opportunities would be great |
| More support from the uni is needed |
| Most of my peers who have been involved in research have used family connections. Other research opportunities that have been mentioned require far to many commitment hours for a standard medical student.     Many people also get their names on publications requiring very little work because they have familial ties with the author. |
| Mostly is who you know in the industry, you have to be good at networking and actively seeking opportunities |
| mostly time commitments - I feel I am already so extremely busy just keeping on top of general university/medical training to have any time/energy to dedicate to research projects |
| Mostly workload from university and inflexibility of curriculum |
| My current University does not have sufficient resources and connections to fund and carry out higher impact and scale research project. Lack of funding, and little focus on research makes it quite inaccessible therefore I have to look for opportunities in other Universities or National/International Associations. |
| My lack of interest |
| my medical school schedules a plethora of useless seminars, workshops and clinical experiences which waste time  that would be better spent on endeavors such as research time |
| My mental health means getting through the degree alone is hard enough, also quite difficult and a lot of effort to find opportunities to do so |
| My university had given little to no guidance on how to obtain research, it's very much a thing you have to do on your own. If we had been educated about research I think I would have fewer barriers. |
| Need to know people who are willing and interested in supervising. |
| Needing to work part time to support myself financially |
| Networking opportunities - it is difficult to know where to begin when looking for opportunities |
| Never been explained, I do not understand how to get into research or what I would need to do |
| Never had an opportunity or met a doctor on the wards involved in research |
| no access to it |
| No allocated time for research  Lack of support and access to supervisors  Lack of funding |
| no exposure to research and how to get involved |
| no formal training |
| no idea how to do it |
| No idea how to get involved? |
| No idea where to access information |
| No idea where to start or who to go to for advice or support. Not feeling qualified/ educated enough. I don't feel I have time alongside medical degree to undertake extra work. |
| No information from the medical school |
| No major barriers but mainly time |
| No major barriers just time around a medical degree for research doesn’t really exist and our Medical School has not talked about research opportunities at this stage so a lot of work would have to go in to finding out how to get involved |
| No mentors |
| No much time to conduct research outside of the medical curriculum |
| No opportunities |
| No personal connections or family members involved with research. Not sure where to start |
| No time |
| No time because of lectures/ working on my degree |
| no time, no money, no connection, not enough resources, not enough confidence in approaching professors |
| None available don’t have the contacts |
| None of the topics so far have interested me and feel very computor based and non team based. I prefer a mixture of both actively working in a lab and working with others to research a topic |
| not a barrier per se, just no incentive or motivation to get involved |
| Not a lot of emphasis on academic research opportunities for medical students |
| not being aware of research opportunities for medical students in early years of education and also the difficulty finding such opportunities |
| Not clear on how to get started. |
| not easy to access research opportunities if you are an undergrad student with no previous research experience |
| Not enough availability for research projects and a lot of the times previous knowledge needed so hard to go into research starting off with limited knowledge |
| not enough exposure or guidance regarding research here at NMS. |
| Not enough information :( - don’t know how to go about finding opportunities |
| not enough information about how to axquire a research opportunity and not enough opportunities |
| Not enough information about scientific writing and how to get published. |
| Not enough information available on how to join one, what they entail |
| Not enough knowledge on how to undertake and publish research- require more teaching and guidance from the university |
| Not enough opportunities |
| Not enough opportunities |
| Not enough opportunities and also partially that the degree itself is so demanding with various deadlines, so sometimes it’s difficult to balance the two |
| Not enough opportunity if not got contacts |
| Not enough opputunities |
| Not enough research opportunities   Time consuming for medical students   Complicated |
| Not enough resources available, difficulty in finding opportunities, not knowing what project and researcher is good |
| Not enough support |
| not enough support from the uni |
| Not enough time |
| Not enough time |
| not enough time during placement work  need more information on how to join |
| Not enough time in medical school |
| Not enough time. No connections to the right people |
| Not given us much direction about how to get involved in research |
| not having any connections or opportunities which would allow me to get involved in research |
| Not having any contacts about how or where to access research supervisors |
| not having any contacts and not knowing who to contact |
| not having the network to ask around for good opportunities. |
| not having the opportunity  not knowing how to take the opportunity if it arises |
| Not interested |
| Not involved in our course as it is elsewhere. Don’t feel confident enough with what we’ve learnt to seek opportunities outside of uni |
| Not knowing how everything works     Lack of experience makes it difficult to start as many roles require prior experience - very frustrating     Lack of connections in the research world |
| not knowing how to get involved |
| Not knowing how to get involved  Not having enough time outside of medical school |
| Not knowing how to get involved, not having the right contacts, not having time to research into how to get into research or knowing how relevant it is to our CV for future jobs etc |
| Not knowing how to get started and how to get published. Not having any personal connections |
| Not knowing how to start |
| Not knowing how to use SPSS  Not having to as a compulsory part of the course  Not enough opportunities to work with clinicians |
| Not knowing how to. I’ve done research in the humanities from my first degree, but it’s hard to get into medical research or know how to start and what it involves as a graduate with research experience in a totally disparate field. I feel like my medical school tells us about research as a tick box exercise eg very little support for coming up with SSC research ideas, no help making contacts to get involved in research etc |
| Not knowing how, not knowing who to ask, not knowing how and where to submit papers, not knowing how to write them |
| Not knowing people doing research so not being offered to be involved in |
| Not knowing where to look for further opportunities or how to get involved |
| Not knowing where to start |
| Not knowing where to start And how to access resources |
| Not knowing where to start looking  Not knowing how to communicate findings |
| Not knowing where to start or who to approach. Not knowing how to process collected data correctly. |
| Not knowing where to start  Feeling intimidated by those already well published |
| Not knowing who to contact |
| Not knowing who to contact for research  Not understanding what roles I can take as a medical student  Never being exposed to research prior to my intercalated dissertation   Finding time to carry out research along with studies and supporting myself financially through medical school  The impression that research is for the select few and arranged through nepotism |
| Not knowing who to contact/ where to start.  Limited time |
| Not knowing who to go to for help |
| Not many opportunities presented to first years. I feel like there will be more chances to partake in reaseach as I advance through medical school |
| Not motivated enough lol |
| Not much information on where to find research opportunitites |
| Not much support |
| Not really as such but the university didn’t give us as many talks on how to actively go about conducting research in the clinical setting - how to go about and find the opportunities |
| Not really sure how |
| Not really sure how to get into research in the first place. Haven’t really been taught how to write a paper or carry out case reports/letters to the editor. And even when I do try to get involved in a project nothing really comes out of it in the end. |
| Not sure how many opportunities there are for first years, but I have not encountered any yet |
| Not sure how to get involved |
| Not sure how to get involved in research. |
| Not sure how to get involved with no experience |
| Not sure how to get involved. |
| Not sure how to get involved/don’t have time |
| Not sure I know enough about it |
| Not sure what opportunities are out there and don’t know where to begin |
| Not sure where to start or who to contact or what I would be able to do |
| not sure where to start, not sure about the process, felt like I didn't have the skills for it |
| Often a lack of clear sign-posting of what projects I can be involved in. |
| Only barrier is papers that require payment to access |
| Only my one pack of knowledge as to how to engage with research |
| Opportunities |
| Opportunities  Amount of time it takes |
| Opportunities and lack of awareness |
| Opportunities are not readily made available. Very hard to conduct research alongside usual med school commitments. |
| Opportunities for research (E.g. Audits, reviews) are not readily publicised to students unless actively sought out. Often students don’t know who to approach to do further research |
| Opportunities not advertised through the medical school as much as they could be |
| Opportunities- we aren’t told Where we can access it |
| Opportunities, time and resources. |
| Opportunity. Access - feeling like I will contribute as opposed to being a burden as a result of my inexperience. |
| Our medical school does not value research and is very, if at all, poorly taught |
| Our teaching is not great for providing neither an interest in pursuing research not the skills required to do so. |
| Outreach and opportunities. Balancing a degree and using this within specific time constraints |
| Pandemic, course demands limit taking on a basic science project |
| People with terrible attitudes looking down on students like me thinking they're better than everyone else |
| Personal barriers such as not feeling confident and not knowing where to look for research opportunities, and in general not being completely interested in undertaking research to be honest |
| Politics within academia |
| Poor background knowledge No clear pathway for seeking opportunities Time commitment required is usually unclear Motivation behind research often unclear (why is this important?) |
| Poor timetableing/ scheduling information from the medical school means I am unable to estimate when I will be free each week/ how much time I would be able to give to research. |
| Previous experience, contacts, direction of research and interests |
| Probably the time commitment amidst the busyness of medical school! |
| Public speaking presenting results |
| realised too late mentors are pretty much everywhere and very approachable in Norwich /NNUH / Nor research park. Med school teaching of research methods hasn't been the best, I'd rate it as a 50% satisfaction rate, and I'm a final year. I didn't get into the local AFP and I struggled a lot on the station of interview for interpreting an abstract! lots of principles I did try to teach myself but found it hard under interview conditions, with new terms id never used or heard of! i haven't heard any of them, beyond simpler concepts taught at Nor med school, things like HR RR OR, CI etc all made sense but other terms not familiar with. |
| Really hard as a medical student to run a QI project. Found massive resistance from current GP for project as we do not work currently in practice |
| Research can seem quite intimidating at first - I'd like to get involved but: 1. Difficult to know where to start 2. Don't want it to overwhelm my clinical studies |
| Research is very time consuming if undertaken alongside a degree. It can also be difficult to find research opportunities. |
| Research supervisors/academics within the medical school can be difficult to engage with as they often have a pre-selection bias toward students that are more arrogant and willing to please. It can be extremely challenging to find a suitable mentor that is willing to help you undertake research activities - there is really no clear path for most students wanting to get involved in research. I believe there is some form of inferiority directed toward medical students within the academic environment, certainly from my experience of the medical school. |
| Rising costs of clinical school as an international student deterring me from taking 3 years out to do a PhD between intercalating year and clinical years. |
| Scared that I'm not competent enough and don't have the basics to fully understood how to do research. |
| Seeing the available opportunities in my areas of interest. |
| Seems very overwhelming and too hard to conduct in conjunction with current study load and exams.   Hard to know where to start. |
| Some students do not wish to share their contacts. |
| Spending time doing research means less time to do other things like:  - revising for medical school exams, which are worth a large proportion of FPAS points  - working, since student loans do not cover my living costs  - training/matches with a university sports team I am on  - actually taking time to relax in the evenings/weekends |
| Staff are not incentisvised to help and so do not help students publish. There are unequal opportunities because the system is based on favouritism and not merit. The uni does not teach us how to get published only how to literature search there is no practical teaching |
| Staff lack of engagement. Lack of contacts. |
| Starting is the hardest as there’s no prerequisite skill |
| Subject not given much focus during teaching, expected to complete research with little time to do it. They assume that we have a good level of preexisting knowledge required to complete the projects and provide little help/opportunity for feedback once given it. There is also limited opportunity to complete research in an area of interest, instead we were asked to rank specialisms of interest and then were assigned project topics somewhat randomly |
| Supervisors  No time in attention to medical school and wanting a life that isn't ONLY medicine. Can't do medicine, extra-curric and research easily. Only way round it seems to be to make medicine your extra-curric |
| Takes too much effort Is it worthwhile if you’re not going t be published? |
| The biggest barrier is networking or finding appropriate research connections. You need to identify relevant contacts informally and approach them yourself, and try and make a good enough impression that they will give you an opportunity. A lot of people don't know how to do this, or find this difficult despite their high academic performance (for example, people with autism or from families that don't have anyone in professional or academic jobs). I would much prefer if student projects were advertised widely to all students in a cohort and the selection process was fairer. |
| the commitment necessary to medical school makes it difficult to take part in research |
| The exposure given by the medical school to conferences, research meetings etc |
| The high workload made it difficult to get time for research, and covid disrupted it further |
| The lack of opportunities available or awareness of how to get involved in these opportunities |
| The medical school provides no background knowledge to approaching research or provide contacts to facilitate opportunities. You have to be very proactice |
| The need to complete medicine |
| The opportunity never presents itself. Never been asked to get involved. |
| The pandemic limited our participants as we could not reach a bigger target so we had to resolve to online surveys. |
| The part of the country in which my clinical years has been based has very little research opportunities |
| The strength of the actual support is not enough |
| The time it takes |
| The workload is extremely high so I just don’t have the time to think about research at the moment. |
| There are not many opportunities |
| There are some SSC as part of our degree but is mainly focused on literature review. I hope to get some opportunity to do lab workk. |
| There aren’t enough opportunities for medical students to conduct research that I know of. |
| There is a lack of a network connecting researchers with students to undertake research. |
| There is a lack of teaching and information to tell medical students about research opportunities or how to get involved in them, by the medical schools. |
| There is barely any information regarding research. To become an adequate doctor you need to take part in clinical audits, write research papers, be published in journals. It would be brilliant if there was some way of being exposed to this kind of level of writing. Just to be a co- author, to get your name on some part of research would be fantastic, but there is no opportunity at all. Conferences are great, but what is the point if you have no data to present, no paper to speak about? There needs to be a little more support and 'push' from the medical schools to encourage students to start writing a little bit of research. The schools need to support students that want to write papers- maybe by them doing a lot of the work and the student inputting a little bit of information in a published article. This would provide the student with relevant exposure and experience. There needs to be more done. |
| There is no explanation given on how to ingage with research or on the different levels of participation, and how these relate to future careers |
| There is no obvious platform that would showcase the opportunities related to research. |
| there is not enough time outside of revising for assessments to take part in research |
| There isn’t as many opportunities to do research as I would like and it really is about who you know when it comes to getting involved. |
| Time |
| Time |
| Time |
| Time |
| Time |
| Time |
| Time |
| time |
| Time |
| Time |
| Time |
| Time |
| Time |
| Time |
| Time - as a grad student on an undergrad degree all my time outside of compulsory classes/placements is spent working to earn money to pay £37k fees |
| Time - balancing exams and research   Lack of supervisors   Insufficient knowledge on how to conduct research   Peers are uninterested |
| Time - have to work to fund medical school. Now that the extra points for research have been removed, there is less incentive to prioritise this. |
| Time - I'm a single parent and have to manage my time very carefully. I struggle as it is juggling parenting with uni work/placements so finding time to fit in research is almost impossible. |
| Time - it is incredibly time consuming     Opportunities - there is a degree of luck involved in finding research opportunities |
| Time - medical school is too busy and having to work part time |
| Time   Competition |
| Time & effort |
| Time / financial constraints (time that would be used for getting involved with research being diverted to studying or working to earn money) |
| Time allocated to research outside of the medical school curriculum. It is impossible to do all lectures, attend placement, exercise, have a social life, keep a job and have the time to dedicate to research.  I was only able to complete my research projects during COVID lockdowns where I was not attending placement and had more free time. |
| Time allowance |
| Time and contacts |
| Time and cost, interest, workload |
| Time and lack of financial compensation -   Medicine is a full on degree so research often has to be done   at weekends/during holidays, especially in clinical years where you are often on the ward most of the day. This is challenging for me (and many other students in similar positions) as I have a part-time job in order to support myself financially while studying. Some degree of financial compensation for research projects would likely help students get into research. |
| Time and money |
| Time and money |
| Time and money and connections |
| Time and money!!! |
| Time and money.   Usually can get involved but only voluntarily, and it is a time-commitment that takes away time from studying or socialising, particularly when also looking for a part-time job. |
| Time and no support |
| time and opportunities |
| time and stress from my degree. I do not want to take part in research if it is at a detriment to qualifying and become a safe and effective clinician |
| Time and understanding of how to get involved |
| Time availability to juggle between medical revisions and research committments. |
| Time available during medical school |
| Time available in medical school, access to pubmed ID journals |
| Time available with my schedule, lack of advertised opportunities |
| Time available with the degree  Guidance on where to find this |
| Time available  Awareness of how to get involved (e.g. I'd love to do an audit but have no idea where to start/where to find info on how to do one)  Lack of contacts/don't know who to contact to get involved  I don't know what I can best fit around my degree without it taking up too much time |
| Time barriers and learning difficulties e.g. dyslexia |
| Time basically - I find doing research is one of the most time-intensive extracurricular activities to have! But would love to have space in my job plan to do it in future. |
| Time commitment - fear that research may be very consuming and not achieve any publishable results |
| time commitment   saturation of poor quality projects, and little mentorship in research |
| Time commitment to clinical placement. Not knowing who to contact to get involved with research. |
| Time commitment to research needed |
| Time commitments |
| Time commitments; as a mature students my time is split between study and family commitments. |
| Time committment required while in medical school. Finding supervisors willing to take on medical students. Getting appropriate recognition on published works. |
| Time constraints |
| Time constraints |
| Time constraints due to work and study |
| Time constraints during a graduate degree |
| Time constraints of balancing medical education with getting involved in research |
| Time constraints of studying medicine |
| Time constraints on a graduate course |
| Time constraints with placements, not knowing how to get involved i.e. who to contact |
| time constraints no paid opportunities no previous publications - the vicious circle of no experience required to get more experience |
| Time consuming |
| Time consuming! Also hard to find the opportunities |
| Time- I’m struggling to keep up with part time work alongside placement, I’ve got like 5 research projects on the go but I have no time to do anything with them |
| Time investment required + outcome is never certain |
| Time involved away from other commitments. Money to finance the cost of travel to collect data |
| Time is limited in Medical School |
| Time is money. The time taken to undertake research, with potentially no reward other personal edification isn’t enough for me. I’m a busy man and I have to carefully select what takes up my time, for me, research isn’t worth it. |
| Time it takes alongside a demanding course |
| Time limitations   Mental health problems |
| Time limits, finding appropriate research projects, gaining benefit from it  E.g. my dissertation supervisor from intercalation said she would look to publish my research, I am yet to hear back from her and it has been months despite me chasing. Feels like supervisors maybe don't want students to publish even if they are keen |
| Time management. Not wanting to sacrifice my grades for time I could spend on research projects.   Knowing which research project I want to do, and how long the process will take to get it into action.   Knowing whether the research will be publishable.   Funding for projects that I could do over the summer months, and knowing where to go |
| Time outside of medicine and hobbies |
| Time pressure. I don’t feel I want to use my little spare time to get involved in research. We have at least 1 research assignment a year that we must complete, however it is “mock” research, so the time I spend on these means I don’t get involved.  I also haven’t had many opportunities presented to me to get involved, but then I also haven’t been actively looking for them. |
| Time pressures |
| Time taken, finding relevant and interesting research projects, not know where to start |
| Time this year. |
| Time to be involved  Feeling adequate to do task  Motivation / interest in topic |
| Time to conduct research.    Research skills not taught in medical school |
| Time to do research alongside medical school studies + placement |
| Time to do so |
| Time to get involved. A lack of awareness about how to get involved with meaningful and useful research. A lack of education about how to establish my own research project. |
| Time to participate in research alongside studies |
| Time  Awareness of opportunities |
| Time  Connections   No clear understanding of the processes |
| Time  Consuming     feel undervalued within a team |
| Time  Disability (fatigue being a main factor) |
| Time  Lack of knowledge in how to get involved |
| Time  Needing to earn money so having less feee time  Few doctors willing to take on extra med student teaching |
| Time  The registrars being too busy to reply to messages during the end stages of research |
| Time Lack of information and availability of opportunity |
| Time Opportunities to get involved not made clear/accessible  Have heard of some friends being involved in research and not credited for their input |
| Time Pressure and inadequate information on the opportunities available and the methodology of research |
| Time Skills |
| Time! |
| Time! Medical students are busy and the timetable is packed |
| Time!! There is already so much to do |
| time, and advertised opportunities within medical school |
| Time, could be earning money in that time, PIs expecting medical students to know far more than we do & then not being supportive when we need assistance which leads to projects getting dropped after lots of work |
| Time, funding, work-life balance, often crazy application / entry requirements / pre-requisite requests |
| Time, interest |
| Time, knowing how/what to access |
| time, knowledge |
| Time, money, contacts and links |
| Time, requires a lot of time which I don't have as I have to work |
| Time, we are expected to be on placement full time unpaid and study on top of this and write coursework, leaving little time for further research |
| Time. Finding the balance between being a clinical medical student and having time to get involved in research can be challenging. In our medical student, with the exception of our compulsory iBSc year, there is no time set aside for research. It is something you have to create for yourself which can be difficult. |
| Time. Just began placement just still getting my bearings |
| Time. Perceived effort to outcome/benefit doesn’t seem to match |
| Time. Very little flexibility around a medical degree to take part in research, I feel that were I to get involved in research, I would be jeopardising my degree. |
| too busy with lectures and revision |
| Too early for me to do anything I think (1st yr) |
| Too much time to get involved, not enough support to find research opportunities that are likely to be successful. No gain from getting involved in research. |
| Transport (and the financial means to pay for it myself)  Free time |
| Tried to help with an orthopaedic study but could not as needed a smart card to access patient records |
| trying to make contacts through covid, gaining experience |
| Unaware of how to get involved and what role I could play. Grants and scholarships or research projects often require prior experience that I don't have. Lack of confidence. |
| Unaware of the motivations why I would, and as I am unfamiliar it'd take me a long time, and due to med school being busy I don't have enough spare time to justify getting involved with research |
| Understanding of clearly defined roles a student can inhabit and what those entail |
| university workload |
| University workload does not allow for spending extra time in research |
| Unsure about how to get into research |
| Unsure how to get involved, don’t have contacts   No clear interests |
| Unsure how to get published and how to conduct original research |
| Unsure of how to get involved |
| Unsure of how to get opportunities or where to start |
| Unsure of the practicalities of doing research   Unsure or who to contact to get involved in research |
| Unsure of where to start, difficulty finding mentors. We no longer have a personal tutor system so that makes it even harder to find a point of contact. With COVID we also don’t see many lecturers in person and aren’t able to form connections |
| Unsure where to really go for quality improvement projects |
| Unsure where to start |
| Unsure who to contact to get involved. No opportunities presented in med school. And unsure if I have research skills to contribute to a team. |
| Very busy with curriculum stuff and travelling to placement and back |
| Very competitive to get into and my school grades suffered due to mental health so I was denied grants/research opportunities on this basis |
| Very difficult to find a supervisor, most are hospital - based and don’t really work with the medical school. Even finding a supervisor for the MRES was a very difficult and independent task. |
| Very difficult to obtain lab projects outside of intercalating, during which time labs were wary about offering projects during the pandemic. Also, time constraints add to the difficulty. |
| Very few opportunities in medical school to approach individuals and get involved in research |
| We aren’t taught how to get involved in projects and where to start. It’s good we have the foundations from the material we are taught about different types of research and how to do it but I think the tricky part is getting into a project. |
| We aren’t usually told when projects are happening locally to get involved with |
| We're not told how to do research |
| We've never been taught how to carry out or structure research so it is quite daunting to try to start doing it |
| Who to contact for specific topics you want to do  Where to start |
| workload |
| Workload and not enough time |
| Worry about written research |
| Would like more advice on how to get involved |
| Wouldn’t know who to contact to get it started. |
| Wouldn't even know where to begin. Seems to be about networking and degree-based opportunities. |
| Yes not enough extra time as I have a part-time job |
| Yes  Funding problems  Inadequate support from the university |
| Yes! My main barrier is the right mentorship and guidance. I didn’t know where to look for projects and what to look for in a GOOD supervisor. I’ve been taken advantage a lot by supervisors |
| Yes, time.  Access to opportunities-awareness  Proper guidance on how to write etc |
| You have to know the right people which can be frustrating |

| **Why did/do you do research?** |
| --- |
| A part of the course |
| As part of my masters degree - compulsory |
| As part of the degree programme |
| Assessed as part of the course |
| Compulsary |
| Compulsory |
| compulsory |
| Compulsory |
| Compulsory |
| Compulsory |
| Compulsory |
| compulsory as part of course |
| Compulsory for BSc |
| Compulsory for FHS |
| Compulsory for the course |
| compulsory on our course |
| Compulsory part of degree |
| Compulsory part of degree |
| Compulsory part of degree |
| Compulsory part of degree |
| Compulsory part of uni degree |
| Compulsory requirement for university degree |
| Compulsory. Some done pre-medical degree |
| course requirement |
| Coursework |
| Data collection was integrated into student selected module |
| Development toward specialty recruitment |
| Dissertation |
| dissertation |
| Don't do any |
| for course |
| For my degree |
| For the patients– I want things (eg services) to be better for them and more fit for purpose |
| Had to |
| had to |
| had to do an audit as part of medical course |
| Had to do it |
| Had to do it for degree |
| Had to do it for the BMedSci |
| Had to/ compulsory bsc |
| Have nothing else to do |
| Haven't |
| Haven't done any |
| Haven't taken any research |
| I didn’t |
| I don’t |
| I don't research. |
| I enjoy it |
| I find it relaxing |
| I had to |
| I had to as part of my course |
| I have not been able to but have an interest in research for intellectual stimulation, my own interest, betterment of healthcare and society and to meet criteria for specialty applications. |
| I haven't yet |
| I knew the doctor doing the audit and wanted to help them. |
| I liked the research team members as mentors |
| Improve student experience |
| Intercalation degree project |
| It is also a tickbox exercise for surgical specialties |
| It was a mandatory part of my course |
| It was a mandatory part of my course |
| it was a required part of my degree |
| It was compulsary |
| it was compulsory |
| it was compulsory |
| It was compulsory |
| It was compulsory part of the course |
| It’s part of my degree |
| It's sadly part of medical school |
| Mandatory Intercalated Degree Project |
| Mandatory part of course |
| Na |
| None |
| none |
| None |
| None |
| Not doing research |
| Only do the compulsory work |
| Part of course |
| Part of course |
| part of course |
| Part of course |
| Part of curriculum |
| part of degree |
| Part of degree |
| Part of Degree |
| Part of intercalated BA |
| Part of intercalated BSc |
| Part of my assesment |
| Part of my course |
| Part of my course |
| Part of my course |
| Part of my course |
| Part of my degree |
| Part of my degree |
| Part of my degree |
| Part of my Masters |
| Part of our course |
| part of our degree |
| Part of our first year ssc |
| Part of previous degree |
| part of school course |
| Part of SSC project |
| Part of the course |
| Part of the course |
| Part of the course |
| Part of the course |
| part of the course |
| Part of the curriculum |
| Part of the degree |
| Part of the intercalation |
| Points for applications / CV |
| Projects part of degree course |
| QUALITY IMPROVEMENT WAS A MANDATORY PROJECT |
| required by course |
| Required for course/degree |
| Required part of my degree |
| Required part of the degree |
| research module as part of course compulsory |
| Special study unit |
| SSC |
| SSC project |
| SSM |
| To gain experience |
| to give evidence to what had been reported anecdotally |
| To prepare for my specialty training |
| Uni work |
| Was always a requirement |
| was required for my degree but I would be interested in doing more research in the future - especially if it leads to publication |
|  |
