# Supplementary material for: Cross-sectional Survey of Medical student perceptions of And desires for Research and Training pathways (SMART): an analysis of prospective cohort study of UK medical students
Source: BMC Med Educ. 2023 Dec 15;23:964. doi: 10.1186/s12909-023-04881-2 (PMC10725016; doi:10.1186/s12909-023-04881-2)
Supplement: Supplementary file 6 — Additional file 6: Appendix S6. Logistic regression results. [file 12909_2023_4881_MOESM6_ESM.docx]

Appendix S6 – Logistic regression results.

|  | | **Univariate ordinal logistic regression** | | **Multivariable ordinal logistic regression** | |
| --- | --- | --- | --- | --- | --- |
|  |  | **OR (95% CI)** | **P value** | **OR (95% CI)** | **P value** |
| **Medical School has well educated the individual about research** | | | | | |
| Year of medical school  (1 = Reference) | 2 | 0.917 (0.698 – 1.205) | 0.534 | NA | NA |
|  | 3 | 0.793 (0.604 – 1.039) | 0.093 | NA | NA |
|  | 4 | 0.791 (0.598 – 1.047) | 0.101 | NA | NA |
|  | 5 | 0.759 (0.560 – 1.028) | 0.074 | NA | NA |
| Type of degree  (Undergraduate = Reference) | Post-graduate | 1.135 [0.876 – 1.470] | 0.337 | NA | NA |
| Completed an academic degree  (No = Reference) | Yes | 1.274 [1.068 – 1.520] | 0.007 | 1.040 [0.850 – 1.273] | 0.700 |
| Ethnicity  (White = Reference) | Asian/Asian British | 0.881 [0.730 – 1.064] | 0.188 | NA | NA |
|  | Black/African/Caribbean/Black British | 0.697 [0.461 – 1.054] | 0.087 | NA | NA |
|  | Mixed/Multiple ethnic groups | 0.746 [0.497 – 1.122] | 0.159 | NA | NA |
|  | Other ethnic group | 0.683 [0.469 – 0.996] | 0.048 | 0.825 [0.536 – 1.270] | 0.382 |
| Gender  (Male = Reference) | Female | 1.012 [0.845 – 1.213] | 0.893 | NA | NA |
|  | Non-binary/ third gender | 1.539 [0.688 – 3.442] | 0.294 | NA | NA |
| LGBTQ  (No = Reference) | Yes | 0.946 [0.750 – 1.193] | 0.640 | NA | NA |
| Eligible for free meals at school  (No = Reference) | Yes | 0.965 [0.745 – 1.251] | 0.789 | NA | NA |
| Per first degree relative who has been a healthcare professional | | 1.088 [1.012 – 1.169] | 0.022 | 1.100 [1.014 – 1.193] | 0.021 |
| Per first degree relative who has been in academia | | 1.058 [0.998 – 1.122] | 0.060 | NA | NA |
| Per first degree relative who has held an academic position in the healthcare environment | | 0.933 [0.863 – 1.008] | 0.080 | NA | NA |
| Area where they undertook the majority of their pre-university education  (UK = Reference) | EU | 0.878 [0.595 – 1.295] | 0.511 | NA | NA |
|  | Outside the EU | 0.588 [0.463 – 0.748] | <0.001 | 0.566 [0.423 – 0.757] | <0.001 |
| Research compulsory part of a degree  (No = Reference) | Yes | 2.092 [1.649 – 2.654] | <0.001 | 2.014 [1.572 -2.581] | <0.001 |
| **Has undertaken research to date** | | | | | |
| Year of medical school  (1 = Reference) | 2 | 1.042 (0.777 – 1.398) | 0.784 | NA | NA |
|  | 3 | 2.001 (1.493 – 2.681) | < 0.001 | 2.001 [1.476 – 2.713] | < 0.001 |
|  | 4 | 2.421 (1.794 – 3.269) | < 0.001 | 1.887 [1.377 – 2.585] | < 0.001 |
|  | 5 | 3.153 (2.288 – 4.343) | < 0.001 | 2.284 [1.609 – 3.242] | < 0.001 |
| Type of degree  (Undergraduate = Reference) | Post-graduate | 1.689 [1.289 – 2.213] | <0.001 | 1.060 [0.769 – 1.462] | 0.721 |
| Completed an academic degree  (No = Reference) | Yes | 2.948 [2.447 – 3.551] | <0.001 | 3.161 [2.500 – 3.997] | <0.001 |
| Ethnicity  (White = Reference) | Asian/Asian British | 1.098 [0.904 – 1.334] | 0.347 | NA | NA |
|  | Black/African/Caribbean/Black British | 1.053 [0.689 – 1.609] | 0.811 | NA | NA |
|  | Mixed/Multiple ethnic groups | 1.119 [0.753 – 1.662] | 0.579 | NA | NA |
|  | Other ethnic group | 1.648 [1.114 – 2.437] | 0.012 | 1.822 [1.190 – 2.790] | 0.006 |
| Gender  (Male = Reference) | Female | 0.830 [0.689 – 1.000] | 0.050 | NA | NA |
|  | Non-binary/ third gender | 2.240 [0.954 – 5.263] | 0.064 | NA | NA |
| LGBTQ  (No = Reference) | Yes | 0.986 [0.775 – 1.254] | 0.909 | NA | NA |
| Eligible for free meals at school  (No = Reference) | Yes | 0.875 [0.667 – 1.147] | 0.334 | NA | NA |
| Per first degree relative who has been a healthcare professional | | 1.088 [1.011 – 1.172] | 0.025 | 1.044 [0.963 – 1.131] | 0.296 |
| Per first degree relative who has been in academia | | 1.040 [0.980 – 1.103] | 0.192 | NA | NA |
| Per first degree relative who has held an academic position in the healthcare environment | | 1.018 [0.938 – 1.106] | 0.666 | NA | NA |
| Area where they undertook the majority of their pre-university education  (UK = Reference) | EU | 1.695 [1.128 – 2.549] | 0.011 | 2.334 [1.484 – 3.669] | <0.001 |
|  | Outside the EU | 1.512 [1.182 – 1.934] | 0.001 | 1.813 [1.354 – 2.428] | <0.001 |
| Research compulsory part of a degree  (No = Reference) | Yes | 1.154 [0.887 – 1.501] | 0.286 | NA | NA |
| How well medical school has educated the individual about research  (Extremely inadequate = Reference) | Somewhat inadequate | 1.169 [0.792 – 1.726] | 0.431 | NA | NA |
|  | Neither adequate nor inadequate | 1.676 [1.124 – 2.500] | 0.011 | 1.940 [1.258 – 2.993] | 0.003 |
|  | Somewhat adequate | 2.449 [1.678 – 3.574] | <0.001 | 2.787 [1.853 – 4.191] | <0.001 |
|  | Extremely adequate | 5.059 [3.234 – 7.914] | <0.001 | 5.822 [3.576 – 9.479] | <0.001 |
| **Finds research useful in combination with medical studies** | | | | | |
| Year of medical school  (1 = Reference) | 2 | 0.682 [0.517 – 0.899] | 0.007 | 0.708 [0.535 – 0.936] | 0.015 |
|  | 3 | 0.545 [0.414 – 0.718] | <0.001 | 0.530 [0.399 – 0.702] | <0.001 |
|  | 4 | 0.563 [0.426 – 0.744] | <0.001 | 0.529 [0.397 – 0.703] | <0.001 |
|  | 5 | 0.610 [0.451 – 0.825] | 0.001 | 0.561 [0.410 – 0.768] | <0.001 |
| Type of degree  (Undergraduate = Reference) | Post-graduate | 1.059 [0.816 – 1.374] | 0.668 | NA | NA |
| Completed an academic degree  (No = Reference) | Yes | 1.183 [0.993 – 1.408] | 0.059 | NA | NA |
| Ethnicity  (White = Reference) | Asian/Asian British | 1.026 [0.850 – 1.238] | 0.789 | NA | NA |
|  | Black/African/Caribbean/Black British | 0.971 [0.641 – 1.470] | 0.888 | NA | NA |
|  | Mixed/Multiple ethnic groups | 0.820 [0.556 – 1.210] | 0.317 | NA | NA |
|  | Other ethnic group | 0.932 [0.644 – 1.349] | 0.710 | NA | NA |
| Gender  (Male = Reference) | Female | 1.169 [0.975 – 1.400] | 0.091 | NA | NA |
|  | Non-binary/ third gender | 1.068 [0.456 – 2.501] | 0.880 | NA | NA |
| LGBTQ  (No = Reference) | Yes | 0.952 [0.754 -1.201] | 0.679 | NA | NA |
| Eligible for free meals at school  (No = Reference) | Yes | 1.146 [0.881 – 1.489] | 0.310 | NA | NA |
| Per first degree relative who has been a healthcare professional | | 1.021 [0.949 – 1.099] | 0.572 | NA | NA |
| Per first degree relative who has been in academia | | 1.003 [0.948 – 1.060] | 0.923 | NA | NA |
| Per first degree relative who has held an academic position in the healthcare environment | | 0.942 [0.870 – 1.019] | 0.135 | NA | NA |
| Area where they undertook the majority of their pre-university education  (UK = Reference) | EU | 1.978 [1.326 – 2.951] | 0.001 | 1.760 [1.143 – 2.710] | 0.010 |
|  | Outside the EU | 1.320 [1.034 – 1.684] | 0.026 | 1.268 [0.981 – 1.637] | 0.069 |
| Research compulsory part of a degree  (No = Reference) | Yes | 1.061 [0.838 – 1.344] | 0.622 | NA | NA |
| How well medical school has educated the individual about research  (Extremely inadequate = Reference) | Somewhat inadequate | 0.763 [0.516 – 1.129] | 0.176 | NA | NA |
|  | Neither adequate nor inadequate | 0.820 [0.550 – 1.222] | 0.329 | NA | NA |
|  | Somewhat adequate | 0.973 [0.667 – 1.419] | 0.888 | NA | NA |
|  | Extremely adequate | 1.718 [1.106 – 2.670] | 0.016 | 1.659 [1.032 – 2.667] | 0.037 |
| Research undertaken to date  (None at all = Reference) | A little | 1.076 [0.859 – 1.348] | 0.522 | NA | NA |
|  | A moderate amount | 1.593 [1.235 – 2.053] | <0.001 | 1.564 [1.180 – 2.073] | 0.002 |
|  | A lot | 2.356 [1.570 – 3.537] | <0.001 | 1.911 [1.229 – 2.973] | 0.004 |
|  | A great deal | 5.116 [2.708 – 9.664] | <0.001 | 3.768 [1.837 – 7.730] | <0.001 |
| **Finds combining research with medical studies difficult** | | | | | |
| Year of medical school  (1 = Reference) | 2 | 0.754 [0.564 – 1.007] | 0.056 | 0.836 [0.620 – 1.127] | 0.239 |
|  | 3 | 0.675 [0.505 – 0.903] | 0.008 | 0.797 [0.589 – 1.079] | 0.142 |
|  | 4 | 0.674 [0.502 – 0.906] | 0.009 | 0.759 [0.554 – 1.040] | 0.087 |
|  | 5 | 0.730 [0.529 – 1.006] | 0.054 | 0.878 [0.621 – 1.242] | 0.462 |
| Type of degree  (Undergraduate = Reference) | Post-graduate | 0.654 [0.495 – 0.862] | 0.003 | 0.713 [0.517 – 0.984] | 0.040 |
| Completed an academic degree  (No = Reference) | Yes | 0.808 [0.671 – 0.973] | 0.024 | 0.767 [0.607 – 0.969] | 0.026 |
| Ethnicity  (White = Reference) | Asian/Asian British | 1.155 [0.946 – 1.410] | 0.157 | NA | NA |
|  | Black/African/Caribbean/Black British | 0.965 [0.613 – 1.520] | 0.879 | NA | NA |
|  | Mixed/Multiple ethnic groups | 0.800 [0.528 – 1.214] | 0.294 | NA | NA |
|  | Other ethnic group | 1.038 [0.695 – 1.550] | 0.857 | NA | NA |
| Gender  (Male = Reference) | Female | 0.718 [0.592 – 0.870] | 0.001 | 0.700 [0.570 – 0.859] | 0.001 |
|  | Non-binary/ third gender | 0.278 [0.115 – 0.675] | 0.005 | 0.250 [0.096 – 0.651] | 0.005 |
| LGBTQ  (No = Reference) | Yes | 0.855 [0.668 – 1.094] | 0.212 | NA | NA |
| Eligible for free meals at school  (No = Reference) | Yes | 0.859 [0.648 -1.137] | 0.288 | NA | NA |
| Per first degree relative who has been a healthcare professional | | 1.056 [0.980 – 1.138] | 0.150 | NA | NA |
| Per first degree relative who has been in academia | | 1.061 [0.999 – 1.126] | 0.052 | NA | NA |
| Per first degree relative who has held an academic position in the healthcare environment | | 1.046 [0.967 – 1.132] | 0.261 | NA | NA |
| Area where they undertook the majority of their pre-university education  (UK = Reference) | EU | 0.936 [0.618 – 1.418] | 0.754 | NA | NA |
|  | Outside the EU | 0.999 [0.773 – 1.293] | 0.997 | NA | NA |
| Research compulsory part of a degree  (No = Reference) | Yes | 1.228 [0.955 – 1.579] | 0.109 | NA | NA |
| How well medical school has educated the individual about research  (Extremely inadequate = Reference) | Somewhat inadequate | 1.336 [0.887 – 2.010] | 0.165 | NA | NA |
|  | Neither adequate nor inadequate | 2.159 [1.420 – 3.284] | <0.001 | 1.937 [1.235 – 3.036] | 0.004 |
|  | Somewhat adequate | 2.388 [1.606 – 3.550] | <0.001 | 2.082 [1.362 – 3.184] | 0.001 |
|  | Extremely adequate | 6.050 [3.797 – 9.639] | <0.001 | 4.767 [2.885 – 7.878] | <0.001 |
| Research undertaken to date  (None at all = Reference) | A little | 0.992 [0.778 – 1.265] | 0.951 | NA | NA |
|  | A moderate amount | 1.695 [1.289 – 2.230] | <0.001 | 1.442 [1.053 – 1.974] | 0.022 |
|  | A lot | 2.185 [1.410 – 3.385] | <0.001 | 1.607 [0.987 – 2.615] | 0.056 |
|  | A great deal | 1.397 [0.725 – 2.695] | 0.318 | NA | NA |
| How useful research is in combination with medical studies (Not at all useful = Reference) | Slightly useful | 1.482 [0.889 – 2.470] | 0.131 | NA | NA |
|  | Moderately useful | 2.476 [1.497 – 4.094] | <0.001 | 2.308 [1.302 – 4.090] | 0.004 |
|  | Very useful | 2.696 [1.622 – 4.480] | <0.001 | 2.552 [1.433 – 4.543] | 0.001 |
|  | Extremely useful | 3.435 [1.983 – 5.952] | <0.001 | 2.873 [1.544 – 5.346] | 0.001 |
| **Wants to pursue an academic career** | | | | | |
| Year of medical school  (1 = Reference) | 2 | 0.811 [0.620 – 1.062] | 0.128 | 1.096 [0.772 – 1.556] | 0.609 |
|  | 3 | 0.629 [0.481 – 0.822] | 0.001 | 0.854 [0.612 – 1.193] | 0.355 |
|  | 4 | 0.770 [0.586 – 1.012] | 0.061 | 1.045 [0.745 – 1.465] | 0.800 |
|  | 5 | 0.599 [0.444 – 0.808] | 0.001 | 0.727 [0.508 – 1.041] | 0.082 |
| Type of degree  (Undergraduate = Reference) | Post-graduate | 1.192 [0.921 – 1.541] | 0.181 | NA | NA |
| Completed an academic degree  (No = Reference) | Yes | 1.146 [0.965 – 1.361] | 0.120 | NA | NA |
| Ethnicity  (White = Reference) | Asian/Asian British | 1.349 [1.121 – 1.624] | 0.002 | 1.308 [1.022 – 1.675] | 0.033 |
|  | Black/African/Caribbean/Black British | 1.061 [0.703 – 1.602] | 0.778 | NA | NA |
|  | Mixed/Multiple ethnic groups | 0.880 [0.594 – 1.303] | 0.523 | NA | NA |
|  | Other ethnic group | 1.236 [0.857 – 1.783] | 0.257 | NA | NA |
| Gender  (Male = Reference) | Female | 1.037 [0.868 – 1.239] | 0.687 | NA | NA |
|  | Non-binary/ third gender | 1.158 [0.523 – 2.564] | 0.717 | NA | NA |
| LGBTQ  (No = Reference) | Yes | 0.966 [0.767 – 1.216] | 0.767 | NA | NA |
| Eligible for free meals at school  (No = Reference) | Yes | 1.306 [1.011 – 1.688] | 0.041 | 1.353 [0.982 – 1.864] | 0.065 |
| Per first degree relative who has been a healthcare professional | | 0.951 [0.886 – 1.022] | 0.172 | NA | NA |
| Per first degree relative who has been in academia | | 1.021 [0.964 – 1.081] | 0.486 | NA | NA |
| Per first degree relative who has held an academic position in the healthcare environment | | 0.953 [0.886 – 1.025] | 0.193 | NA | NA |
| Area where they undertook the majority of their pre-university education  (UK = Reference) | EU | 1.860 [1.244 – 2.782] | 0.003 | 1.609 [0.976 – 2.653] | 0.062 |
|  | Outside the EU | 1.335 [1.054 – 1.692] | 0.017 | 1.097 [0.807 – 1.491] | 0.554 |
| Research compulsory part of a degree  (No = Reference) | Yes | 0.758 [0.600 – 0.959] | 0.021 | 0.744 [0.574 – 0.964] | 0.025 |
| How well medical school has educated the individual about research  (Extremely inadequate = Reference) | Somewhat inadequate | 1.061 [0.733 – 1.537] | 0.753 | NA | NA |
|  | Neither adequate nor inadequate | 0.963 [0.660 – 1.407] | 0.847 | NA | NA |
|  | Somewhat adequate | 1.066 [0.746 – 1.523] | 0.727 | NA | NA |
|  | Extremely adequate | 1.109 [0.725 – 1.695] | 0.633 | NA | NA |
| Research undertaken to date  (None at all = Reference) | A little | 0.988 [0.793 – 1.230] | 0.912 | NA | NA |
|  | A moderate amount | 1.436 [1.119 – 1.844] | 0.004 | 1.246 [0.988 – 1.570] | 0.063 |
|  | A lot | 2.722 [1.801 – 4.114] | <0.001 | 2.091 [1.368 – 3.196] | 0.001 |
|  | A great deal | 5.287 [2.827 – 9.887] | <0.001 | 2.596 [1.280 – 5.267] | 0.008 |
| How useful research is in combination with medical studies (Not at all useful = Reference) | Slightly useful | 2.367 [1.438 – 3.896] | 0.001 | 2.121 [1.142 – 3.940] | 0.017 |
|  | Moderately useful | 9.927 [6.021 – 16.368] | <0.001 | 3.934 [2.136 – 7.244] | <0.001 |
|  | Very useful | 9.927 [6.021 – 16.368] | <0.001 | 9.266 [4.979 -17.244] | <0.001 |
|  | Extremely useful | 37.736 [21.853 – 65.164] | <0.001 | 30.202 [15.3-5 – 59.600] | <0.001 |
| Difficulty of combining research with medical studies (Extremely difficult = Reference) | Somewhat difficult | 1.274 [1.005 – 1.615] | 0.046 | 1.303 [0.961 – 1.766] | 0.089 |
|  | Neither easy nor difficult | 1.147 [0.871 – 1.511] | 0.329 | NA | NA |
|  | Somewhat easy | 1.297 [0.893 – 1.884] | 0.172 | NA | NA |
|  | Extremely easy | 3.539 [1.485 – 8.435] | 0.004 | 1.464 [0.558 – 3.840] | 0.438 |
| **Wants to pursue an academic training pathway** | | | | | |
| Year of medical school  (1 = Reference) | 2 | 0.764 [0.585 – 0.998] | 0.048 | 0.907 [0.688 – 1.198] | 0.492 |
|  | 3 | 0.573 [0.438 – 0.749] | <0. 001 | 0.687 [0.518 – 0.911] | 0.009 |
|  | 4 | 0.689 [0.523 – 0.907] | 0.008 | 0.848 [0.635 – 1.134] | 0.267 |
|  | 5 | 0.393 [0.289 – 0.533] | <0.001 | 0.454 [0.329 – 0.627] | <0.001 |
| Type of degree  (Undergraduate = Reference) | Post-graduate | 1.370 [1.056 – 1.778] | 0.018 | 1.384 [1.045 – 1.833] | 0.023 |
| Completed an academic degree  (No = Reference) | Yes | 1.108 [0.932 – 1.317] | 0.244 | NA | NA |
| Ethnicity  (White = Reference) | Asian/Asian British | 1.312 [1.091 – 1.578] | 0.004 | 1.296 [1.046 – 1.604] | 0.018 |
|  | Black/African/Caribbean/Black British | 1.017 [0.673 – 1.538] | 0.935 | NA | NA |
|  | Mixed/Multiple ethnic groups | 0.892 [0.604 – 1.317] | 0.565 | NA | NA |
|  | Other ethnic group | 1.118 [0.772 – 1.620] | 0.556 | NA | NA |
| Gender  (Male = Reference) | Female | 0.919 [0.769 – 1.097] | 0.347 | NA | NA |
|  | Non-binary/ third gender | 0.747 [0.323 – 1.728] | 0.495 | NA | NA |
| LGBTQ  (No = Reference) | Yes | 0.950 [0.754 – 1.196] | 0.663 | NA | NA |
| Eligible for free meals at school  (No = Reference) | Yes | 1.284 [0.993 – 1.661] | 0.056 | NA | NA |
| Per first degree relative who has been a healthcare professional | | 0.98 [0.917 – 1.056] | 0.651 | NA | NA |
| Per first degree relative who has been in academia | | 0.992 [0.938 – 1.048] | 0.773 | NA | NA |
| Per first degree relative who has held an academic position in the healthcare environment | | 0.974 [0.905 – 1.047] | 0.474 | NA | NA |
| Area where they undertook the majority of their pre-university education  (UK = Reference) | EU | 2.167 [1.457 – 3.221] | <0.001 | 1.608 [1.031 – 2.508] | 0.036 |
|  | Outside the EU | 1.289 [1.015 – 1.635] | 0.037 | 1.114 [0.848 – 1.464] | 0.437 |
| Research compulsory part of a degree  (No = Reference) | Yes | 0.876 [0.693 – 1.108] | 0.270 | NA | NA |
| How well medical school has educated the individual about research  (Extremely inadequate = Reference) | Somewhat inadequate | 0.999 [0.691 – 1.444] | 0.996 | NA | NA |
|  | Neither adequate nor inadequate | 1.018 [0.698 – 1.485] | 0.925 | NA | NA |
|  | Somewhat adequate | 1.122 [0.786 – 1.599] | 0.527 | NA | NA |
|  | Extremely adequate | 1.235 [0.809 – 1.885] | 0.329 | NA | NA |
| Research undertaken to date  (None at all = Reference) | A little | 0.932 [0.750 – 1.159] | 0.527 | NA | NA |
|  | A moderate amount | 1.378 [1.075 – 1.767] | 0.012 | 1.339 [1.008 – 1.778] | 0.044 |
|  | A lot | 2.636 [1.732 – 4.012] | <0.001 | 2.365 [1.485 – 3.766] | <0.001 |
|  | A great deal | 5.648 [2.925 -10.903] | <0.001 | 4.185 [1.899 – 9.223] | <0.001 |
| How useful research is in combination with medical studies (Not at all useful = Reference) | Slightly useful | 2.012 [1.239 – 3.269] | 0.005 | 1.957 [1.145 – 3.347] | 0.014 |
|  | Moderately useful | 3.408 [2.113 – 5.498] | <0.001 | 2.984 [1.755 – 5.074] | <0.001 |
|  | Very useful | 6.493 [3.996 – 10.550] | <0.001 | 5.463 [3.186 – 9.367] | <0.001 |
|  | Extremely useful | 20.916 [12.328 – 35.486] | <0.001 | 14.624 [8.158 – 26.214] | <0.001 |
| Difficulty of combining research with medical studies (Extremely difficult = Reference) | Somewhat difficult | 1.464 [1.153 – 1.860] | 0.002 | 1.372 [1.058 – 1.779] | 0.017 |
|  | Neither easy nor difficult | 1.470 [1.113 – 1.941] | 0.007 | 1.287 [0.950 – 1.744] | 0.103 |
|  | Somewhat easy | 1.941 [1.339 – 2.813] | <0.001 | 1.099 [0.734 – 1.646] | 0.646 |
|  | Extremely easy | 4.121 [1.678 – 10.119] | 0.002 | 2.290 [0.886 – 5.918] | 0.087 |
| **Interested in undertaking more research in the future** | | | | | |
| Year of medical school  (1 = Reference) | 2 | 0.851 [0.645 – 1.123] | 0.255 | 1.188 [0.821 – 1.720] | 0.361 |
|  | 3 | 0.703 [0.534 – 0.927] | 0.012 | 0.998 [0.699 – 1.426] | 0.993 |
|  | 4 | 1.084 [0.821 – 1.433] | 0.569 | 1.776 [1.229 – 2.566] | 0.002 |
|  | 5 | 0.948 [0.699 – 1.284] | 0.728 | 1.236 [0.835 – 1.828] | 0.290 |
| Type of degree  (Undergraduate = Reference) | Post-graduate | 1.075 [0.825 – 1.401] | 0.593 | NA | NA |
| Completed an academic degree  (No = Reference) | Yes | 1.317 [1.105 – 1.570] | 0.002 | 1.194 [0.945 – 1.508] | 0.138 |
| Ethnicity  (White = Reference) | Asian/Asian British | 0.924 [0.765 – 1.115] | 0.409 | NA | NA |
|  | Black/African/Caribbean/Black British | 0.967 [0.636 – 1.468] | 0.873 | NA | NA |
|  | Mixed/Multiple ethnic groups | 0.826 [0.559 – 1.220] | 0.336 | NA | NA |
|  | Other ethnic group | 0.873 [0.593 – 1.284] | 0.490 | NA | NA |
| Gender  (Male = Reference) | Female | 1.215 [1.014 – 1.457] | 0.035 | 1.345 [1.068 – 1.694] | 0.012 |
|  | Non-binary/ third gender | 0.634 [0.289 – 1.394] | 0.257 | NA | NA |
| LGBTQ  (No = Reference) | Yes | 1.043 [0.827 – 1.317] | 0.720 | NA | NA |
| Eligible for free meals at school  (No = Reference) | Yes | 1.011 [0.775 – 1.318] | 0.936 | NA | NA |
| Per first degree relative who has been a healthcare professional | | 0.957 [0.892 – 1.026] | 0.213 | NA | NA |
| Per first degree relative who has been in academia | | 0.939 [0.889 – 0.992] | 0.025 | 0.912 [0.850 – 0.979] | 0.011 |
| Per first degree relative who has held an academic position in the healthcare environment | | 0.930 [0.863 – 1.002] | 0.058 | NA | NA |
| Area where they undertook the majority of their pre-university education  (UK = Reference) | EU | 2.562 [1.687 – 3.892] | <0.001 | 2.132 [1.258 – 3.614] | 0.005 |
|  | Outside the EU | 1.499 [1.174 – 1.913] | 0.001 | 1.276 [0.943 – 1.727] | 0.114 |
| Research compulsory part of a degree  (No = Reference) | Yes | 0.687 [0.538 – 0.876] | 0.002 | 0.638 [0.480 – 0.848] | 0.002 |
| How well medical school has educated the individual about research  (Extremely inadequate = Reference) | Somewhat inadequate | 0.672 [0.455 – 0.992] | 0.046 | 0.780 [0.462 – 1.315] | 0.350 |
|  | Neither adequate nor inadequate | 0.516 [0.346 – 0.771] | 0.001 | 0.660 [0.386 – 1.128] | 0.129 |
|  | Somewhat adequate | 0.645 [0.441 – 0.943] | 0.024 | 0.677 [0.409 -1.120] | 0.129 |
|  | Extremely adequate | 0.729 [0.467 – 1.138] | 0.164 | NA | NA |
| Research undertaken to date  (None at all = Reference) | A little | 0.951 [0.761 – 1.189] | 0.661 | NA | NA |
|  | A moderate amount | 1.337 [1.037 – 1.723] | 0.025 | 1.168 [0.912 – 1.497] | 0.219 |
|  | A lot | 1.521 [1.002 – 2.309] | 0.049 | 0.994 [0.636 – 1.555] | 0.980 |
|  | A great deal | 2.904 [1.511 – 5.583] | 0.001 | 1.292 [0.580 – 2.877] | 0.531 |
| How useful research is in combination with medical studies (Not at all useful = Reference) | Slightly useful | 4.408 [2.644 – 7.349] | <0.001 | 3.805 [1.995 – 7.258] | <0.001 |
|  | Moderately useful | 10.817 [6.497 – 18.011] | <0.001 | 8.600 [4.532 – 16.318] | <0.001 |
|  | Very useful | 30.852 [18.266 – 52.108] | <0.001 | 23.871 [12.377 – 46.039] | <0.001 |
|  | Extremely useful | 127.233  [70.382 – 230.005] | <0.001 | 88.214 [41.962 – 185.448] | <0.001 |
| Difficulty of combining research with medical studies (Extremely difficult = Reference) | Somewhat difficult | 1.114 [0.877 – 1.416] | 0.376 | NA | NA |
|  | Neither easy nor difficult | 0.932 [0.701 – 1.239] | 0.630 | NA | NA |
|  | Somewhat easy | 1.082 [0.744 – 1.575] | 0.680 | NA | NA |
|  | Extremely easy | 1.655 [0.685 – 4.002] | 0.263 | NA | NA |
| **Feels there are barriers preventing them from getting involved in research** | | **Univariate logistic regression** | | **Multivariable ordinal logistic regression** | |
|  |  | **OR (95% CI)** | **P value** | **OR (95% CI)** | **P value** |
| Year of medical school  (1 = Reference) | 2 | 1.292 [0.946 – 1.764] | 0.107 | 1.697 [1.093 – 2.636] | 0.018 |
|  | 3 | 1.600 [1.176 – 2.177] | 0.003 | 1.984 [1.299 – 3.028] | 0.002 |
|  | 4 | 1.795 [1.308 – 2.463] | <0.001 | 2.432 [1.580 – 3.743] | <0.001 |
|  | 5 | 1.978 [1.406 – 2.781] | <0.001 | 2.776 [1.764 – 4.369] | <0.001 |
| Type of degree  (Undergraduate = Reference) | Post-graduate | 0.844 [0.629 – 1.132] | 0.258 | NA | NA |
| Completed an academic degree  (No = Reference) | Yes | 1.072 [0.882 – 1.302] | 0.485 | NA | NA |
| Ethnicity  (White = Reference) | Asian/Asian British | 0.866 [0.702 – 1.069] | 0.181 | NA | NA |
|  | Black/African/Caribbean/Black British | 1.056 [0.663 – 1.682] | 0.819 | NA | NA |
|  | Mixed/Multiple ethnic groups | 1.185 [0.768 – 1.828] | 0.444 | NA | NA |
|  | Other ethnic group | 0.917 [0.601 – 1.399] | 0.688 | NA | NA |
| Gender  (Male = Reference) | Female | 1.926 [1.567 – 2.366] | <0.001 | 1.968 [1.496 – 2.590] | <0.001 |
|  | Non-binary/ third gender | 2.139 [0.872 – 5.246] | 0.097 | NA | NA |
| LGBTQ  (No = Reference) | Yes | 0.975 [0.754 – 1.260] | 0.846 | NA | NA |
| Eligible for free meals at school  (No = Reference) | Yes | 1.285 [0.962 – 1.716] | 0.090 | NA | NA |
| Per first degree relative who has been a healthcare professional | | 0.845 [0.773 – 0.923] | <0.001 | 0.902 [0.788 – 1.031] | 0.130 |
| Per first degree relative who has been in academia | | 0.890 [0.832 – 0.951] | 0.001 | 0.928 [0.839 – 1.026] | 0.145 |
| Per first degree relative who has held an academic position in the healthcare environment | | 0.851 [0.768 – 0.944] | 0.002 | 0.896 [0.767 – 1.048] | 0.170 |
| Area where they undertook the majority of their pre-university education  (UK = Reference) | EU | 1.328 [0.856 – 2.058] | 0.205 | NA | NA |
|  | Outside the EU | 1.278 [0.977 – 1.671] | 0.074 | NA | NA |
| Research compulsory part of a degree  (No = Reference) | Yes | 0.716 [0.550 – 0.931] | 0.013 | 0.723 [0.526 – 0.993] | 0.045 |
| How well medical school has educated the individual about research  (Extremely inadequate = Reference) | Somewhat inadequate | 0.685 [0.441 -1.065] | 0.093 | NA | NA |
|  | Neither adequate nor inadequate | 0.344 [0.219 – 0.539] | <0.001 | 0.567 [0.301 – 1.068] | 0.079 |
|  | Somewhat adequate | 0.304 [0.198 – 0.465] | <0.001 | 0.394 [0.217 – 0.718] | 0.002 |
|  | Extremely adequate | 0.164 [0.098 – 0.274] | <0.001 | 0.314 [0.156 – 0.630] | 0.001 |
| Research undertaken to date  (None at all = Reference) | A little | 0.779 | 0.051 | NA | NA |
|  | A moderate amount | 0.427 | <0.001 | 0.674 [0.507 – 0.898] | 0.007 |
|  | A lot | 0.436 | <0.001 | 0.904 [0.545 – 1.499] | 0.695 |
|  | A great deal | 0.862 | 0.673 | NA | NA |
| How useful research is in combination with medical studies (Not at all useful = Reference) | Slightly useful | 0.907 [0.546 – 1.506] | 0.705 | NA | NA |
|  | Moderately useful | 0.834 [0.507 – 1.372] | 0.475 | NA | NA |
|  | Very useful | 1.154 [0.699 – 1.907] | 0.576 | NA | NA |
|  | Extremely useful | 1.404 [0.817 – 2.413] | 0.219 | NA | NA |
| Difficulty of combining research with medical studies (Extremely difficult = Reference) | Somewhat difficult | 0.356 [0.266 – 0.476] | <0.001 | 0.389 [0.268 – 0.565] | <0.001 |
|  | Neither easy nor difficult | 0.190 [0.135 – 0.268] | <0.001 | 0.239 [0.151 – 0.377] | <0.001 |
|  | Somewhat easy | 0.093 [0.057 – 0.153] | <0.001 | 0.134 [0.072 – 0.248] | <0.001 |
|  | Extremely easy | 0.118 [0.042 – 0.332] | <0.001 | 0.118 [0.031 – 0.450] | 0.002 |
